# The Effects of Graded Levels of Calorie Restriction: XIX. Impact of Graded Calorie Restriction on Protein Expression in the Liver

**DOI:** 10.1093/gerona/glad017

**Published:** 2023-02-09

**Authors:** Lu Wang, Davina Derous, Xiahe Huang, Sharon E Mitchell, Alex Douglas, David Lusseau, Yingchun Wang, John R Speakman

**Affiliations:** School of Pharmacy, Collaborative Innovation Center of Advanced Drug Delivery System and Biotech Drugs in Universities of Shandong, Key Laboratory of Molecular Pharmacology and Drug Evaluation, Ministry of Education, Yantai University, Yantai, China; Institute of Biological and Environmental Sciences, University of Aberdeen, Aberdeen, UK; State Key Laboratory of Molecular Developmental Biology, Institute of Genetics and Developmental Biology, Chinese Academy of Sciences, Chaoyang, Beijing, China; Institute of Biological and Environmental Sciences, University of Aberdeen, Aberdeen, UK; Institute of Biological and Environmental Sciences, University of Aberdeen, Aberdeen, UK; Institute of Biological and Environmental Sciences, University of Aberdeen, Aberdeen, UK; State Key Laboratory of Molecular Developmental Biology, Institute of Genetics and Developmental Biology, Chinese Academy of Sciences, Chaoyang, Beijing, China; Institute of Biological and Environmental Sciences, University of Aberdeen, Aberdeen, UK; State Key Laboratory of Molecular Developmental Biology, Institute of Genetics and Developmental Biology, Chinese Academy of Sciences, Chaoyang, Beijing, China; CAS Centre for Excellence in Animal Evolution and Genetics (CCEAEG), Kunming, China; Shenzhen Key Laboratory of Metabolic Health, Center for Energy Metabolism and Reproduction, Shenzhen Institutes of Advanced technology, Chinese Academy of Sciences, Shenzhen, China

**Keywords:** Carnitine, “Clean cupboard hypothesis”, Hepatic proteome, Major urinary proteins, Metabolic pathways

## Abstract

Calorie restriction (CR) extends life span by modulating the mechanisms involved in aging. We quantified the hepatic proteome of male C57BL/6 mice exposed to graded levels of CR (0%–40% CR) for 3 months, and evaluated which signaling pathways were most affected. The metabolic pathways most significantly stimulated by the increase in CR, included the glycolysis/gluconeogenesis pathway, the pentose phosphate pathway, the fatty acid degradation pathway, the valine, leucine, and isoleucine degradation pathway, and the lysine degradation pathway. The metabolism of xenobiotics by cytochrome P450 pathway was activated and feminized by increased CR, while production in major urinary proteins (Mups) was strongly reduced, consistent with a reduced investment in reproduction as predicted by the disposable soma hypothesis. However, we found no evidence of increased somatic protection, and none of the 4 main pathways implied to be linked to the impact of CR on life span (insulin/insulin-like growth factor [IGF-1], nuclear factor-κB [NF-κB], mammalian Target of Rapamycin [mTOR], and sirtuins) as well as pathways in cancer, were significantly changed at the protein level in relation to the increase in CR level. This was despite previous work at the transcriptome level in the same individuals indicating such changes. On the other hand, we found *Aldh2*, *Aldh3a2*, and *Aldh9a1* in carnitine biosynthesis and *Acsl5* in carnitine shuttle system were up-regulated by increased CR, which are consistent with our previous work on metabolome of the same individuals. Overall, the patterns of protein expression were more consistent with a “clean cupboards” than a “disposable soma” interpretation.

Aging is accompanied by many metabolic changes. Obesity, insulin resistance, inflammation, and high blood pressure are known as the metabolic syndrome of aging (1). One hundred years ago, Osborne and colleagues found female rats stunted by calorie restriction (CR) lived longer (2). Since then, the beneficial effect of CR on longevity has been demonstrated in both sexes of a wide variety of species (3). However, the mechanisms that mediate the beneficial effects of CR on aging are not fully understood, potentially because of the involvement of multiple divergent responses of multiple tissues and pathways (4).

The liver plays a central role in energy metabolism and glucose homeostasis. Previous work suggested that 40% CR changed lipid metabolism in the liver through reduced lipogenesis and increased lipolysis and ketogenesis (5), as well as changes in sphingosine-1-phosphate signaling and carntine biosynthesis and shuttle pathways (6). Short-term CR increases β-fatty acid oxidation, which inhibits triglyceride synthesis, leading to the improvement of non-alcoholic fatty liver disease or hepatic insulin resistance (7). Nicotinamide adenine dinucleotide (NAD) level decreases with age and promotes several aging-associated diseases, including metabolic and neurodegenerative diseases and various cancers. Conversely, increased de novo biosynthesis of NAD or supplementary intake from diet exhibits beneficial effects against aging and related diseases (8). Metabolomic analysis showed that the NAD biosynthesis pathways were up-regulated in multiple tissues including liver when exposed in higher CR grades (4). Xenobiotic metabolism participates in modulation of aging and activation of the genes involved in xenobiotic metabolism has been reported as a shared signature of mouse models with extended life span, including calorically restricted mice (9).

CR was also reported to regulate several canonical aging-related pathways, including insulin/insulin-like growth factor (IGF-1) signaling (10,11), mTOR signaling (12), nuclear factor-κB (NF-κB) signaling (13), and sirtuin signaling (14). Down-regulation of insulin/IGF-1 signaling pathway was associated with increased life span in various species, including rodents (15,16). Inhibition of the mTOR signaling pathway increased the life span of model organisms and protected them against age-related diseases (12), while knocking out the downstream ribosomal protein S6 kinase 1 (S6K1) from mTOR signaling extended the life span in mouse (17). Activation of NF-κB signaling is associated with the insulin/IGF-1 and mTOR signaling pathways, as well as inflammation (13). Up-regulated sirtuin signaling increased longevity in yeasts, fruitflies, and worms (18–20) and was reported to be associated with insulin signaling pathways as well as de novo NAD biosynthesis (21,22), however, the evidence for its importance in aging and longevity in mammals is still weak (23). Finally, CR’s anti-cancer effects may also promote longevity (24). Therefore, in this study, we also looked for changes in insulin/IGF-1, mTOR, NF-B, and Sirtuin signaling pathways, as well as signaling pathways related to cancer, in relation to the increase in the level of CR.

Increased CR in both male and female mice and rats are roughly linearly related to the increase in life span (25,26) up to at least 65% restriction. Hence, using graded levels of CR as a research tool allows one to probe the mechanisms underlying aging and longevity more effectively than exposure to a single level of restriction (27,28). In this study, we used 6 levels of CR treatment in 5-month-old male C57BL/6 mice, restricted for 3 months: 24 hours ad libitum (AL) feeding, 12 hours AL feeding (time-restricted feeding) 10% CR, 20% CR, 30% CR, and 40% CR. Linear changes in protein expression with the level of restriction may be key components underpinning the longevity response. We previously used a correlation approach across different levels of CR to investigate changes in the hepatic transcriptome (29) and metabolome (4,6). Here, we performed similar work in the same individual mice that were analyzed previously using the liver proteome.

## Results

### General Correlation Approach and Enrichment Analyses

**Figure 1.**
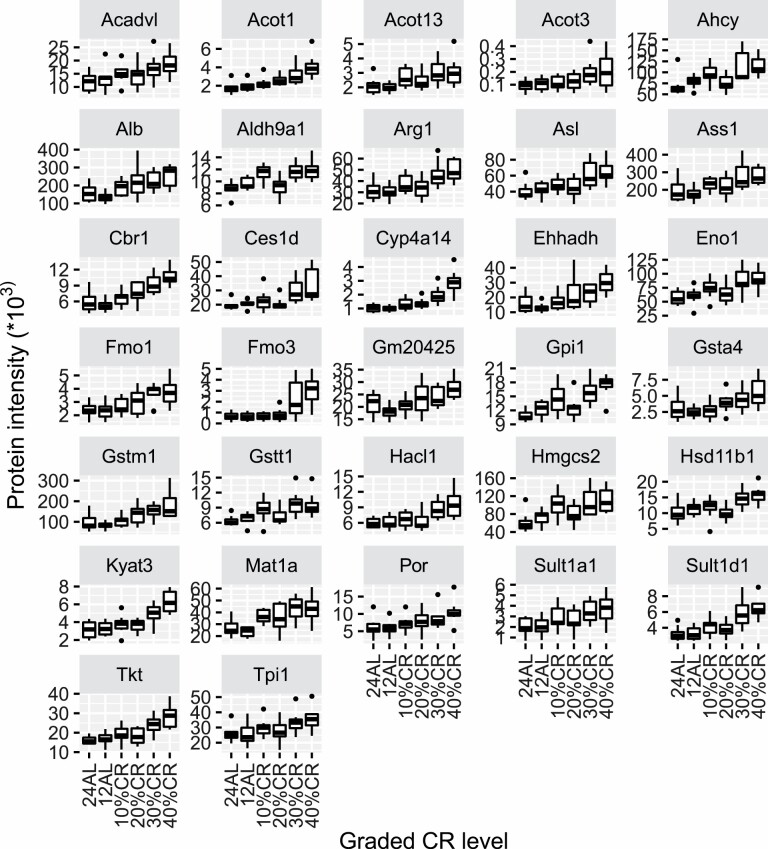
Protein expression of the 32 genes that were positively correlated (adjusted *p* < .05) with the increase of calorie restriction (CR) plotted against the CR level. Protein expression of genes were correlated with the increase in CR level by Pearson correlation method, and *p* values were adjusted by the Benjamini–Hochberg method. The x-axis represents the CR level and the y-axis represents intensity value of the protein expression. 24AL stands for mice being fed ad libitum (AL) for 24 hours a day (*n* = 8); 12AL stands for mice being fed AL for 12 hours a day during the dark period (*n* = 8); 10%CR (*n* = 8), 20%CR (*n* = 8), 30%CR (*n* = 8), and 40%CR (*n* = 8) indicates mice being fed 10%, 20%, 30%, and 40% lower calories respectively than their own individual intakes measured over a baseline period of 14 days prior to introducing CR.

**Figure 2.**
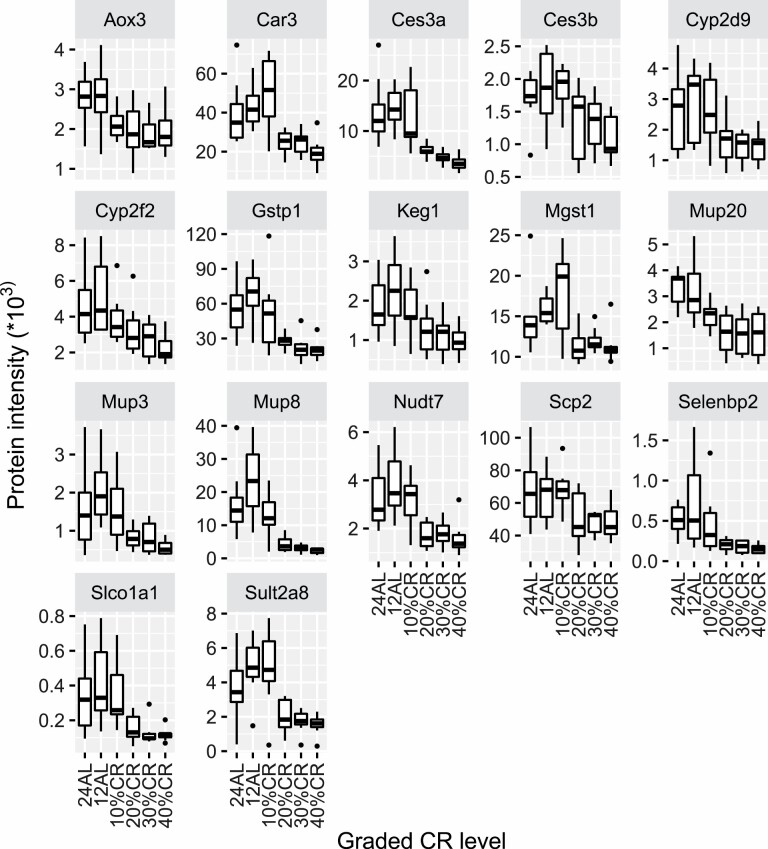
Protein expression of the 17 genes that were negatively correlated (adjusted *p* < .05) with the increase of calorie restriction (CR) plotted against the CR level. Protein expression of genes were correlated with the increase in CR level by Pearson correlation method, and *p* values were adjusted by Benjamini–Hochberg method. The x-axis represents the CR level and the y-axis represents intensity value of the protein expression. 24AL stands for mice being fed ad libitum (AL) for 24 hours a day (*n* = 8); 12AL stands for mice being fed AL for 12 hours a day during the dark period (*n* = 8); 10%CR (*n* = 8), 20%CR (*n* = 8), 30%CR (*n* = 8), and 40%CR (*n* = 8) indicates mice being fed 10%, 20%, 30%, and 40% lower calories, respectively than their own individual intakes measured over a baseline period of 14 days prior to introducing CR.

Protein expressions of a total 2 206 genes from the proteomic data were correlated with the increase in restriction, and 134 correlations were considered statistically significant (*p* < .05). Almost three-quarters (99) of the 134 correlations were positive, meaning the protein expression of a majority of significantly altered genes was up-regulated by the increase in CR level. Forty-nine of the 134 genes remained significant after correction for multiple testing using the Benjamini-Hochberg procedure, with 32 of these positively correlated (Figure 1) and 17 negative (Figure 2). Two-dimensional principal component analysis was performed using all the 2 206 genes’ protein expression of each individual, and the result echoed that the protein expression of the most genes was not changed by the increased CR level (Supplementary Figure 1). Enrichment analysis for the 134 genes found 91 gene ontology (G.O.) terms with adjusted *p* value < .05 and 27 of those 91 terms enriched in more than 10 genes (Supplementary Figure 2), of which “extracellular exosome,” “cytoplasm,” and “cytosol” were the most significant. The enrichment analysis also detected 33 Kyoto Encyclopedia of Genes and Genomes (KEGG) pathways with adjusted *p* value < .05 (Supplementary Figure 3). “Metabolic pathways” was the most significant with the smallest adjusted *p* value (<10^−29^) and the largest number (77) of enriched genes. The majority (63) of the genes enriched in the “metabolic pathways” were up-regulated (Supplementary Table 1). Changes of the “metabolic pathways” were mainly concentrated in carbohydrate, lipid, and amino acid metabolism pathways (Supplementary Table 1).

Linear regression was also performed with the increased CR level as the independent variable and the intensity of protein expression of each gene as the dependent variable, where the graded CR level was transferred into an ordered factor (“24AL” < “12AL” < “10%CR” < “20%CR” < “30%CR” < “40%CR”), and 132 regressions were statistically significant (*p* < .05), 126 out of which overlapped with the correlation results. After adjusting the *p* values for multiple testing, only 45 regressions were statistically significant (adjusted *p* < .05), 44 out of which overlapped with the adjusted correlation results. There were consequently very few differences between the results of the correlation analysis and the linear regression modeling, and therefore the following analyses are based on the correlation results to maintain consistency with the hepatic transcriptome paper we published previously relating to the same individual animals (29).

### Glycolysis Pathway

**Figure 3.**
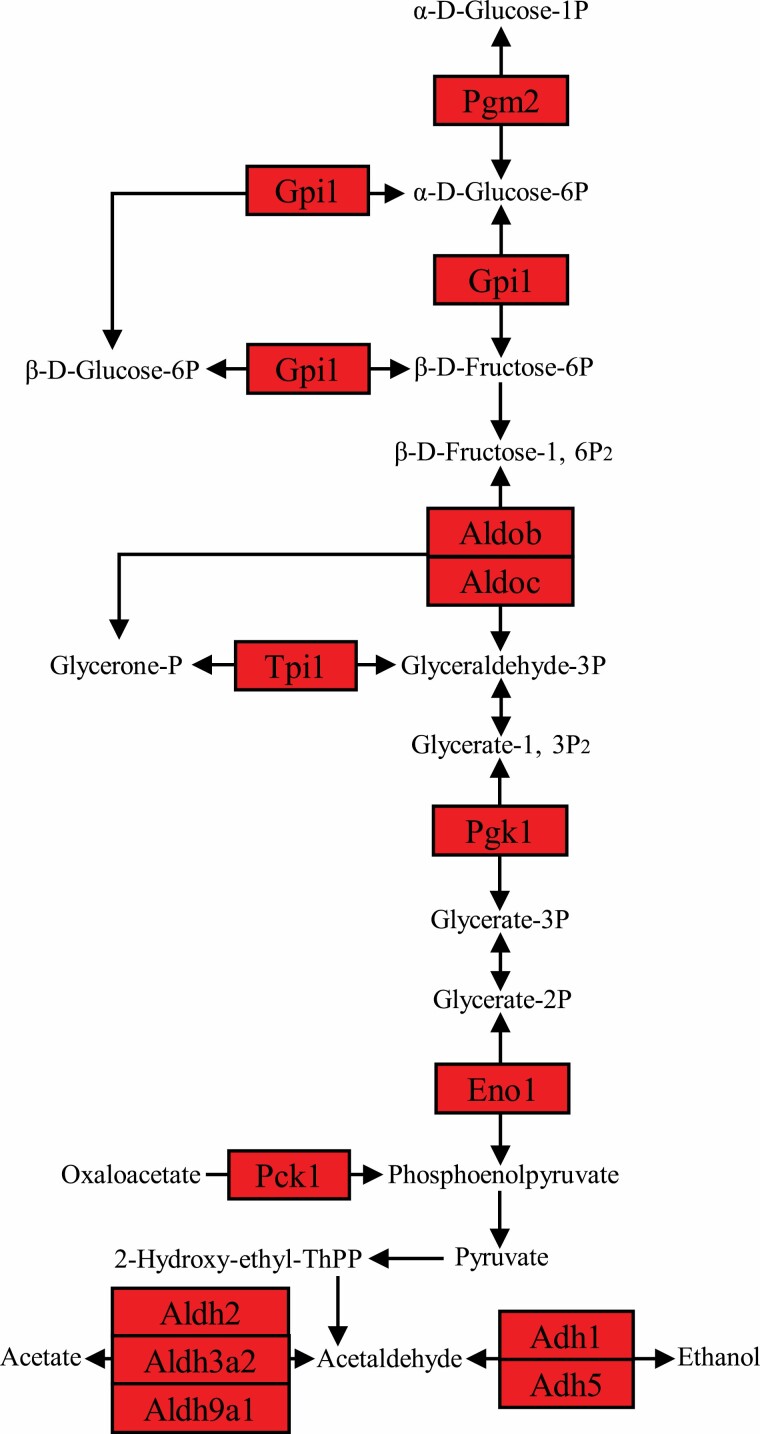
Glycolysis pathway diagram showing Pearson correlation of protein expression of the mapped genes with the increased calorie restriction (CR) level. The pathway was colored based on the correlation coefficients and the *p* values, with red standing for significant (*p* < .05) positive correlation and blue standing for significant (*p* < .05) negative correlation. The pathway diagram was customized based on glycolysis/gluconeogenesis pathway obtained from Kyoto Encyclopedia of Genes and Genomes (KEGG).

The glycolysis pathway was activated in relation to the increasing levels of CR (Figure 3 and Supplementary Figure 4). Phosphoglucomutase 2 (Pgm2) was up-regulated by the increase in CR level (Supplementary Table 1). Pgm2 catalyzes the inter-conversion between glucose-1-phosphate (G-1-P) and glucose-6-phosphate (G-6-P). Expressions of glucose-6-phosphate isomerase 1 (Gpi1), fructose-bisphosphate aldolase B (Aldob) and C (Aldoc), triosephosphate isomerase 1 (Tpi1), phosphoglycerate kinase 1 (Pgk1), enolase 1 (Eno1), and aldehyde dehydrogenase family genes, enzymes involved in the glycolytic pathway, were also significantly up-regulated in parallel with the increase in CR level (Supplementary Table 1). Acetyl-CoA, an important precursor metabolite, is produced by oxidative decarboxylation of pyruvate, and this process was not significantly changed.

### Citrate Cycle (TCA cycle)

The citrate cycle is the key aerobic pathway for the final steps of the oxidation of carbohydrates and fatty acids. Expressions of isocitrate dehydrogenase 1 (Idh1) and 2 (Idh2), and malate dehydrogenase 2 (Mdh2) were significantly up-regulated as the CR level increased (Supplementary Figure 5 and Supplementary Table 1). Isocitrate dehydrogenase catalyzes the oxidative decarboxylation of isocitrate, resulting in alpha-ketoglutarate and carbon dioxide. Malate dehydrogenase catalyzes the reversible transformation of malate into oxaloacetate.

### Pentose Phosphate Pathway

The pentose phosphate pathway was stimulated by increase in the CR level (Supplementary Figure 6). Although there was no significant change in the irreversible oxidative phase of this pathway, the expression of transketolase (Tkt) in the reversible oxidative phase was significantly up-regulated by increased CR (Supplementary Table 1). Phosphoribosyl pyrophosphate (PRPP) is an activated compound used in the biosynthesis of histidine and purine/pyrimidine nucleotides, and the expression of Pgm2, an enzyme involved in the formation of PRPP, was significantly stimulated by increased CR level.

### Glyoxylate and Dicarboxylate Metabolism Pathway

Glyoxalase 1 (Glo1) and Glyoxalase 2 (Glo2) play essential roles in the detoxification of methylglyoxal, and the product of the detoxification, glycolate, is a substrate for glycolate oxidase to produce glyoxylate. There was no significant change in protein expressions of Glo1 and Glo2 with the increased CR level, and in the glyoxylate and dicarboxylate metabolism pathways, only Mdh2 and serine hydroxymethyltransferase 1 (soluble) (Shmt1) proteins were significantly upregulated by the increase in CR (Supplementary Figure 7 and Supplementary Table 1).

### Fatty Acid Metabolism

**Figure 4.**
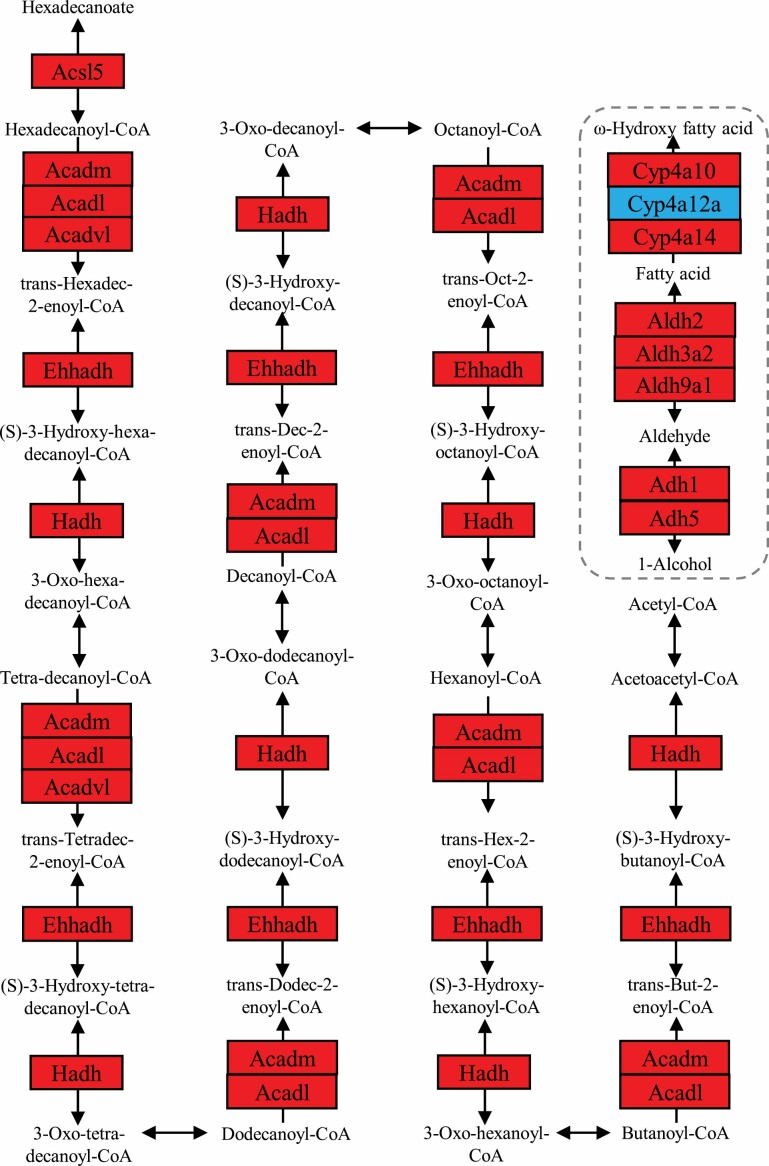
Fatty acid degradation pathway diagram showing Pearson correlation of protein expression of the mapped genes with the increased calorie restriction (CR) level. The pathway was colored based on the correlation coefficients and the *p* values, with red standing for significant (*p* value < .05) positive correlation and blue standing for significant (*p* value < .05) negative correlation. The pathway diagram was customized based on fatty acid degradation pathway obtained from Kyoto Encyclopedia of Genes and Genomes (KEGG).

Fatty acid metabolism, in particular fatty acid oxidation, was stimulated by the increase in CR (Figure 4 and Supplementary Figure 8). Fatty acid metabolism mainly consists of fatty acid elongation and β-oxidation in mitochondria, which are essentially a reversal of each other. Acsl5, the enzyme catalyzing the conversion of long-chain fatty acids to their active form acyl-CoAs for both synthesis of cellular lipids and degradation via beta-oxidation, was up-regulated by the increase in CR, so were the enzymes of β-fatty acid oxidation, including acyl-Coenzyme A dehydrogenase, long chain (Acadl), medium chain (Acadm), and very long chain (Acadvl), as well as Enoyl-Coenzyme A, hydratase/3-hydroxyacyl Coenzyme A dehydrogenase (Ehhadh), and hydroxyacyl-Coenzyme A dehydrogenase (Hadh) (Supplementary Table 1), while expression of mitochondrial trans-2-enoyl-CoA reductase (Mecr; *r* = −0.03692, *p* = .8307) showed no significant correlation to the increased CR level, which indicated that it was the β-oxidation process, aka the degradation pathway, that was stimulated rather than the elongation pathway. Sterol carrier protein 2, liver (Scp2), a nonspecific lipid-transfer protein was significantly inhibited by the increase in CR (Supplementary Table 1).

The carnitine shuttle system transports fatty acids into mitochondria for β-oxidation. Most genes mapped to this system, including carnitine palmitoyl-transferase 1a, liver (Cpt1a), carnitine palmitoyltransferase 2 (Cpt2), solute carrier family 25 (mitochondrial carnitine/acylcarnitine translocase), member 20 (Slc25a20), and acyl-CoA synthetase long-chain family member 1(Acsl1), showed no significant changes in protein expression along with the increase in CR levels, except for Acsl5, which was up-regulated. The carnitine biosynthesis pathway starts from trimethyllysine (obtained from the diet or synthesized from L-lysine) and is part of the pathway of lysine degradation. Aldehyde dehydrogenase 2, mitochondrial (Aldh2), aldehyde dehydrogenase family 3, subfamily A2 (Aldh3a2), and aldehyde dehydrogenase 9, subfamily A1 (Aldh9a1) are included in the pathway of carnitine biosynthesis and their protein expression were significantly up-regulated by the increase in CR (Supplementary Table 1). Sphingolipid metabolite sphingosine-1-phosphate (S1p) regulates cellular responses to stress as a growth and survival factor and was identified to be changed in our previous work on CR effects on liver metabolomics (6). However, both S1P signaling and biosynthesis pathway showed no significant changes in protein expression in relation to the increase in CR.

### PPAR Signaling Pathway

Peroxisome proliferator-activated receptors (PPARs) are nuclear hormone receptors that are activated by fatty acids and their derivatives. However, no protein expression of PPARs was detected from the liver proteome. Protein expression of fatty acid transporter Slc27a5/FATP of the downstream PPAR signaling pathway was significantly up-regulated by the increase in CR level (Supplementary Figure 9 and Supplementary Table 1). Of PPAR targets involved in fatty acid oxidation, Acadl/LCAD, Acadm/MCAD, Ehhadh/Bien, and long-chain fatty acid omega-mono-oxygenase Cyp4a10 and Cyp4a14 were up-regulated, however, Cyp4a12a and Scp2 were down-regulated (Supplementary Figure 9 and Supplementary Table 1). Of PPAR targets participating in fatty acid transport, Acsl5 was up-regulated, while fatty acid binding protein 1, liver (Fabp1; *r* = −0.3436, *p* = .01681) was down-regulated (Supplementary Figure 9). Among other PPARs targeted processes, 3-hydroxy-3-methylglutaryl- CoA synthase 2 (Hmgcs2) involved in ketogenesis was up-regulated, so was malic enzyme 1, NADP(+)-dependent, cytosolic (Me1) involved in lipogenesis and Pck1/PEPCK included in the gluconeogenesis pathway (Supplementary Figure 9).

### Amino Acid Metabolism

Increasing CR level stimulated amino acid biosynthesis pathways mostly because it stimulated the protein expression of genes (Aldob, Aldoc, Eno1, Idh1, Idh2, Pgk1, Tkt, and Tpi1) overlapping with the glycolysis, TCA cycle, and pentose phosphate pathways, which provide activated precursors for biosynthesis of various amino acids, such as histidine. However, cystathionase (cystathionine gamma-lyase) (Cth), enzyme participating in cysteine biosynthesis, was also significantly up-regulated (Supplementary Figure 10 and Supplementary Table 1). Arginase (Arg1), argininosuccinate lyase (Asl), and argininosuccinate synthetase 1 (Ass1), which are involved in arginine biosynthesis as well as the urea cycle, were significantly up-regulated, so were glutamic-oxaloacetic transaminase 1 (Got1), an enzyme that also plays an important role in amino acid metabolism and in the urea and TCA cycles (Supplementary Figures 10 and 11 and Supplementary Table 1).

Serine dehydratase (Sds), the enzyme mediating the conversion from serine to pyruvate, was up-regulated; Methionine adenosyltransferase I, alpha (Mat1a), a liver-specific enzyme that catalyzes the formation of S-adenosylmethionine, the principal biological methyl donor, was also up-regulated; Serine hydroxymethyltransferase 1 (soluble) (Shmt1), an enzyme which plays an important role in cellular one-carbon pathways by catalyzing the conversions of L-serine to glycine and 5,6,7,8-tetrahydrofolate to 5,10-methylenetetrahydrofolate, was also up-regulated (Supplementary Figure 10 and Supplementary Table 1).

Degradation of various amino acids were also activated by the increased CR level. Expressions of aldehyde dehydrogenase 9, subfamily A1 (Aldh9a1), aldehyde dehydrogenase family 3, subfamily A2 (Aldh3a2), aldehyde dehydrogenase 2, mitochondrial (Aldh2), Hmgcs2, Ehhadh, Hadh, and Acadm, enzymes mediating degradation of the branched-chain amino acids (valine, leucine, and isoleucine) were significantly up-regulated (Supplementary Figure 12 and Supplementary Table 1). Expressions of aminoadipate-semialdehyde synthase (Aass), Aldh2, Aldh3a2, Aldh9a1, Ehhadh, and Hadh, enzymes mediating the degradation of Lysine, were also significantly up-regulated (Supplementary Figure 13 and Supplementary Table 1).

In de novo biosynthesis pathways, NAD is generated from tryptophan via 2-amino-3-carboxymuconic-6-semialdehyde and quinolinic acid, which is part of tryptophan metabolism, and genes mapped to the NAD biosynthesis, including tryptophan 2,3-dioxygenase (Tdo2), indoleamine 2, 3-dioxygenase 2 (Ido2), arylformamidase (Afmid), kynurenine 3-monooxygenase (Kmo), and amino carboxymuconate semialdehyde decarboxylase (Acmsd), showed no significant changes in protein levels, except for kynureninase (Kynu), which converts N′-formylkynurenine into kynurenine, down-regulated (*r* = −0.3061, *p* = .03437) by the increased CR.

### Metabolism of Xenobiotics by Cytochrome P450s

**Figure 5.**
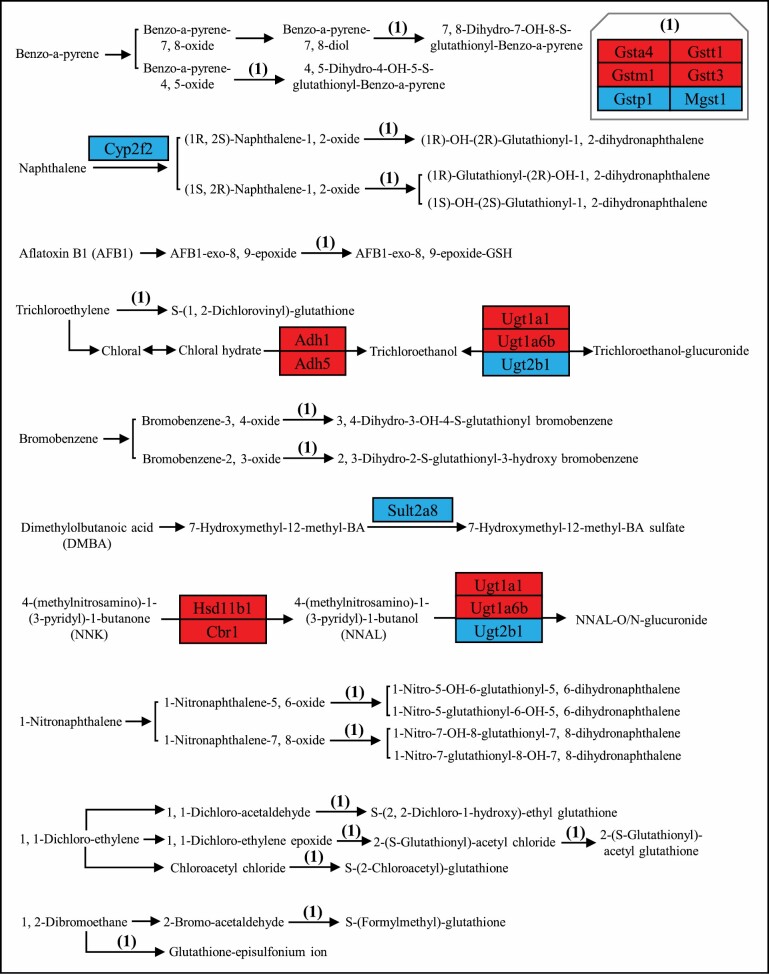
Metabolism of xenobiotics by cytochrome P450 pathway diagram showing Pearson correlation of protein expression of the mapped genes with the increased calorie restriction (CR) level. The pathway was colored based on the correlation coefficients and the *p* values, with red standing for significant (*p* value < .05) positive correlation and blue standing for significant (*p* value < .05) negative correlation. The pathway diagram was customized based on metabolism of xenobiotics by cytochrome P450 pathway obtained from Kyoto Encyclopedia of Genes and Genomes (KEGG).

Metabolism of xenobiotics by cytochrome P450s, a detoxification-associated pathway, was significantly changed by the increased CR level (Figure 5 and Supplementary Figure 14). Alcohol dehydrogenases, Adh1 and Adh5 were up-regulated. Carbonyl reductase 1 (Cbr1) and hydroxysteroid 11-beta dehydrogenase 1 (Hsd11b1) were also up-regulated. Glutathione S-transferases, Gsta4, Gstm1, Gstt1, and Gstt3 were stimulated, while Gstp1 and Mgst1 (microsomal) were down-regulated by the increase in CR. Of Uridine 5′-diphospho-glucuronosyltransferase, Ugt1a1 and Ugt1a6b were activated by the increase in CR level, while Ugt2b1 was inhibited (Supplementary Table 1). Cytochrome P450, family 2, subfamily f, polypeptide 2 (Cyp2f2; *r* = −0.5620, *p* < 10^−4^) and sulfotransferase family 2A, Dehydroepiandrosterone-preferring (DHEA-preferring), member 8 (Sult2a8; *r* = −0.597789705, *p* < 10^−5^) were down-regulated by the increased CR level ( Supplementary Figure 14).

### Longevity Regulating Pathways

Longevity regulating pathways obtained from KEGG consist of 4 pathways that have been implicated in mediating the CR effect on extending life span. These are the insulin/IGF-1 pathway, the SIRT pathway, the AMPK pathway, and the mTOR pathway. In this study, however, no significant correlations was found between the expression of proteins in components of any of these “longevity regulating” pathways and the increase in CR (Supplementary Figures 15–18). We also obtained NF-kappa B signaling pathway from KEGG, another pathway linked to aging, and there was no protein expression related to NF-ĸB signaling pathway that showed a significant correlation to the increased CR level (Supplementary Figures 19 and 20).

### Pathways in Cancer

Pathways in Cancer obtained from KEGG included the Wnt, Hedgehog, Notch, HIF-1, cAMP, mTOR, PI3K-Akt, Jak-STAT, MAPK, Calcium, TGF-β, VEGF, p53, and PPAR signaling pathways, and none of these pathways showed significant changes as the CR level was increased (Supplementary Figure 21). However, the epidermal growth factor receptor (Egfr), enhancing inflammatory cytokine signaling, thereby promoting tumor cell progression, was down-regulated (*r* = −0.3316, *p* = .02132) by the increase in CR. Gstp1 and Mgst1 were also down-regulated, while Gstm1, Gsta4, Gstt1, and Gstt3 were significantly up-regulated. Those are the glutathione S-transferase enzymes, which are the phase II enzymes of the biotransformation enzyme group responsible for detoxification of xenobiotics and protection against cancers.

### Hepatic mRNA and Protein Expression Correlation Across Individuals

In our earlier work concerning the hepatic transcriptome of the same individual animals (29), we found mRNA levels of transcripts in the IGF-1/insulin pathway, mTOR, NF-ĸB, and the sirtuin signaling pathways significantly changed in relation to the increased CR levels (absolute mean correlation coefficient across genes in relation to the restriction level = 0.47, mean *p* = .01). Yet in the present analysis, protein expression of genes in those pathways was almost unaffected by the CR levels (absolute mean correlation coefficient = 0.16, mean *p* = .60, and there was a lack of significant correlation between the protein expression and the mRNA expression (Supplementary Table 2). Therefore, we assessed whether the hepatic mRNA levels corresponded more generally to protein expression.

We identified the top 30 genes correlating positively with the increase in CR at the transcript level, the top 30 negatively correlating genes, and 30 genes where changes were least correlated with the restriction level. For these 90 genes, the transcript levels were correlated with their corresponding protein levels. A significant positive association was found between the transcript levels and protein levels for 47 of the 60 genes where there was a strong treatment effect: both up and down (Supplementary Figure 22). However, when there was no treatment effect on mRNA expression, only 1/30 genes showed a significant correspondence between mRNA and protein levels (Supplementary Figure 22). These data show that the mRNA levels were strongly related to the corresponding protein levels across individuals but only when there was a really strong treatment effect (Supplementary Figure 23).

### Increased CR Feminized Male Mice

The increased CR stimulated genes related to xenobiotic process, including Cyp4a14, P450 oxidoreductase (Por; *r* = 0.49, *p* < .001), flavin containing monooxygenase 3 (Fmo3; *r* = 0.66, *p* < 10^−6^) and Fmo1 (*r* = 0.57, *p* < 10^−4^), sulfotransferase family 1D, member 1 (Sult1d1; *r* = 0.69, *p* < 10^−7^) and Sult1a1 (*r* = 0.50, *p* < .001), and Gsta4. Expression of these genes has previously been shown to be more highly expressed in females in the mouse liver because of the suppressive effects of both androgens and male-pattern growth hormone (GH) secretion (30).

On the other hand, carboxylesterase 3A (Ces3a; *r* = −0.73, *p* < 10^−8^), Ugt2b1, and Gstp1 were down-regulated by the increased CR, which normally have a greater expression in males because of the inducing effect of male-pattern GH secretion (31). Moreover, the expression of mouse major urinary proteins were also inhibited by increased CR levels, which are sex-speciﬁc genes and speciﬁcally expressed in the male mouse liver, including Mup8 (*r* = −0.71, *p* < 10^−7^), Mup20 (*r* = −0.63, *p* < 10^−5^), Mup3 (*r* = −0.57, *p* < 10^−4^), Mup2 (*r* = −0.51, *p* < .01), Mup14 (*r* = −0.47, *p* < .01), and Mup10 (*r* = −0.47, *p* < .01). These changes suggested an increasing feminization in relation to the level of restriction.

## Discussion

The G.O. enrichment of the protein list showed the most intensive enrichment of extracellular exosome, cytoplasm, and cytosol. Exosomes are extracellular vesicles released from cells through multi-vesicular body (32), and it was not surprising to find most proteins involved in G.O. term exosome overlapped with those in cytoplasm and cytosol because the exosome lumen is made of cytosol. Immune cells such as B cells, dendritic cells and mast cells release exosomes continuously or secrete exosomes upon stimulation by cell–cell interactions, and the best-established functions of exosomes is in the immune response (32). Recently, it has been revealed that hepatocyte exosomes from early onset obese mice (4 weeks high-fat diet) express high levels of microRNA miR-3075, which improves insulin sensitivity, while hepatocyte-released exosomes from chronic obese mice (16–18 weeks high-fat diet) promote insulin resistance (33). Another speculated role of exosomes is to maintain cellular homeostasis through the export of excess and unnecessary constituents from cells (34,35). Whether graded CR stimulates hepatic exosome release requires further investigation.

Pathway enrichment analysis showed the most intensive enrichment of the metabolic pathways, including carbohydrate, lipid, and amino acid metabolism pathways. These pathways were all stimulated by increased CR. It has been suggested that suppression of glycolysis and/or activation of pentose phosphate pathway could contribute to the beneficial effects of caloric restriction on aging by decreasing methylglyoxal, one of the most potent glycating agents, and by upregulating proteolysis processes like autophagy (36,37). In this study, the glycolysis pathway and the pentose phosphate pathway were both activated by the increase in CR level. Under normal conditions, the detoxification of methylglyoxal is performed by the glyoxalase system where Glyoxalase 1 (Glo1) and Glyoxalase 2 (Glo2) play essential roles, and the product of the detoxification is glycolate, a substrate for glycolate oxidase to produce glyoxylate (36). However, we found no significant change in protein expressions of Glo1 and Glo2 with the increased CR level, and the glyoxylate and dicarboxylate metabolism pathway was not significantly changed either. This suggests that there was possibly sufficient capacity in this system to deal with elevated methylglyoxal without requiring up-regulation.

Mice on CR exhibited up-regulated fatty acid oxidation, and a shift from carbohydrate metabolism to fatty acid oxidation, which may reduce the production of ROS and reduce oxidative damage, and thereby contribute to the benefit of CR (38). In this study, fatty acid oxidation was activated, with enzymes involved in the initial step of mitochondrial fatty acid β-oxidation, Acadvl, Acadl, and Acadm, all up-regulated by the increased CR level. Acsl5 was also upregulated. This enzyme, involved in the carnitine shuttle system, catalyzes the first step in intracellular fatty acid metabolism: the conversion of fatty acids to acyl-CoA thioesters, which are substrates for both esterified lipid synthesis and β-oxidation. Contrasting our findings this gene was reported to be expressed poorly in the liver during fasting and was strongly induced by increased carbohydrate concentrations and the insulin level (39). However, our previous work on liver metabolome showed that the carnitine shuttle system was up-regulated by the increased CR, which is consistent with the Acsl5 protein expression change in this work. Moreover, Aldh2, Aldh3a2, and Aldh9a1 involved in the biosynthesis of carnitine were upregulated in protein expressions, which was also consistent with the metabolome work (4,6).

The mouse Cyp4a family is an important component of fatty acid metabolism, and in this study, Cyp4a10 and Cyp4a14 were both up-regulated by the increased CR level. It was previously reported that hepatic overexpression of Cyp4a14 increases lipid accumulation in the livers of wild-type mice (40). Previous work also reported that at metabolic steady state, fatty acid oxidation must match fatty acid intake plus synthesis, and during CR fatty acid synthesis was stimulated to make up for the shortfall in intake (38).

Our previous work on liver, brain, and brown adipose tissue metabolomes showed that, across multiple tissues, NAD levels up-regulated by increased CR, which may be related to the beneficial impacts of CR for aging, since NAD levels decrease with age and de novo biosynthesis and dietary supplementation of NAD provides benefits against aging (4,8). However, in this study, in the pathways of de novo NAD biosynthesis from tryptophan via 2-amino-3-carboxymuconic-6-semialdehyde and quinolinic acid, only the gene Kynu, which converts N′-formylkynurenine into kynurenine, was down-regulated, while Tdo2, Ido2, Afmid, Kmo, and Acmsd, showed no significant changes by the increased CR. On the other hand, the pathways of carnitine shuttle system and biosynthesis, critical for fatty acid β-oxidation, were up-regulated by the increased CR, which is consistent with our previous metabolome work (4,6). Acetyl-coenzyme A (acetyl-CoA), a pivotal central intermediate metabolites, showed no significant change in our previous metabolome work (4,6), nor did its synthesis pathways in this proteome work.

The 4 main signaling pathways previously implied to be linked to the impact of CR on life span, the insulin/ IGF-1, NF-ĸB, mTOR, and SIRT signaling pathways, showed no significant changes with the increase in CR level, neither did the pathways in cancer. In our earlier work concerning the hepatic transcriptome of the same mice, we looked at these 4 main signaling pathways and found all the pathways were altered by increased CR levels. This raises broader questions about the relation between gene expressions at the transcript level compared with the protein level. Nevertheless, a significant positive association was found between the mRNA transcript levels and protein levels in the cases where there was a highly significant response (both positive and negative) to the increase in CR level. In contrast, when the mRNA expression levels did not respond significantly to the increase in CR, there was only one out of 30 genes showed a significant correlation between the mRNA transcript levels and protein levels. Even though the mRNA expression of genes in the 4 main longevity-regulating related pathways were significantly correlated (mean *p* = .01) with the increased CR level, there was a lack of significant correlation between the protein expression and level of CR (mean *p* = .60) and a lack of significant correlation between the protein and mRNA expression. These data suggest that mRNA levels are only a good indicator of altered protein expression when the relationship to a given treatment variable is very strong. This is in agreement with a previous study which found that in the livers of mice treated for different periods of time with 3 different peroxisome proliferative-activated receptor agonists, the differential expression of mRNA (up or down) captured at most 40% of the variation of protein expression (41), and recent work which observed distinctive changes in mRNA and protein levels as a function of age in the kidney in mice (42).

Although we found a statistically significant correspondence between mRNA and protein levels when perturbed by CR, the variation between individuals in transcript levels clearly did not explain all of the variations in protein levels. Protein abundance levels are controlled by post-transcriptional, translational, and protein degradation regulation processes (43). Studies have previously tried to unravel the contribution of each of these processes to the protein abundance and found that post-transcription, translation, and protein degradation contribute as much to the variation in protein concentrations as transcription and transcript degradation (43,44). Nevertheless, we observed a clear “treatment effect” on the correspondence between mRNA and protein levels. Further work, potentially involving more sophisticated analytical methods than we have used here, is needed to understand how these different processes contribute to protein levels and their correspondence to transcript levels under different conditions.

The main evolutionary hypothesis for CR’s effects on longevity is the “disposable soma hypothesis (DSH)” (45). The DSH suggests that under the limited energy conditions of CR, animals invest in somatic maintenance at the cost of reproduction. Supporting this idea, we found that males were generally more feminized by increased CR, with some female-dominated gene expressions up-regulated, such as Cyp4a14, and some male-dominated gene expressions down-regulated such as Mups. Since these Mups are secreted into the urine and are a major investment by male mice in marking territories that they defend to monopolize reproductive opportunities with females living in their territories, their down-regulation with increasing CR level is highly consistent with the DSH interpretation of how CR works. Moreover, the downregulation of Mups proteins in the liver is consistent with the reduced levels of Mups in the urine of the same individual mice (10). In contrast, however, we did not find any evidence for the upregulation of protein levels of genes that might be considered diagnostic of somatic protection.

Recently, a novel idea called the “clean cupboards hypothesis” was proposed which suggests that when animals are under CR, they do not strategically reallocate resources between reproduction and somatic protection, but rather their aim is only to make an immediate energy balance (46). Consequently, reducing the production of Mups would also be consistent with this latter idea as well as the DSH. Critically the changes in other pathways we identified, including upregulation of lipid oxidation and elevated amino acid degradation, along with the absence of any evidence for upregulated somatic protection, are more consistent with this latter “clean cupboards hypothesis.”

## Conclusion

The metabolic pathways, including carbohydrate, lipid, and amino acid metabolism pathways were the most significantly stimulated by the increased CR level. There was the significant feminization of these male mice and reduced investment in major urinary proteins. We found no upregulation of pathways that might be considered diagnostic of somatic protection. Moreover, the lipid metabolism modulating pathways carnitine shuttle system and biosynthesis were up-regulated, which is consistent with our previous metabolome work. Several key pathways previously suggested to play an important role in CR’s effect to life span (specifically insulin/growth hormone, mTOR, sirtuin, and NF-ĸB pathways) were virtually unchanged at the protein level, despite previous transcriptomics work in the same individual animals showing significant changes.

## Method

### Animals and Experimental Manipulations

All procedures were approved by the University of Aberdeen ethical approval committee and carried out under the Animals (Scientific Procedures) Act 1986 Home Office license (PPL 60/3706 held by J.R.S.). Forty-eight male C57BL/6 mice (*Mus musculus*) purchased from Charles River (Ormiston, UK) were individually housed with free access to water. Mice were exposed to 12-hour dark/light cycles (lights on at 0630 hours) and body mass and food intake were recorded daily, immediately prior to nocturnal feeding. At 20 weeks of age (resembling early adulthood in humans), mice were randomly allocated into 6 different treatment groups: (12AL *n* = 8, 24AL *n* = 8, 10%CR *n* = 8, 20%CR *n* = 8, 30%CR *n* = 8, 40%CR *n* = 8). Mice in 24AL and 12AL groups were fed AL for 24 hours or 12 hours during the dark period, respectively. 10%CR, 20%CR, 30%CR, and 40%CR indicate 10%, 20%, 30%, and 40% lower calories, respectively, than their own individual intakes measured over a baseline period of 14 days prior to introducing CR.

Animals fed completely AL (ie, having 24 hours of access to food) may overfeed and become obese. CR-associated changes compared to 24AL are, therefore, most likely to reflect the anti-obesity effect of CR (3,47). In addition, CR-restricted mice generally consume food during the first few hours of food provided. The 24AL animals can by definition, eat at any time throughout a 24-hour period. Hence, when CR-restricted mice were culled, they may have been starving for 10–16 hours, while 24AL may have eaten in the hour prior to culling. To address this issue, 12AL was set as a reference to avoid the “time since last meal effect” and graded levels of CR were introduced to investigate a potential graded response. Information on the overall study design, diet composition, and detailed rationale are described elsewhere (48).

### Proteomics

After culling by a terminal CO_2_ overdose, the liver was removed as part of the overall dissection, weighed, and divided into 7 pieces which were immediately snap frozen in liquid nitrogen and stored at −80°C until one piece was used for proteome isolation. The proteins were digested using the filter-aided proteome sample preparation method by sequencing grade trypsin (Promega, Madison, WI). The resultant tryptic peptides from each sample were then labeled with the 8-plex iTraq-reagents (AB Sciex, Inc., Framingham, MA). The labeled samples were mixed together with an equal molar ratio. After prefrcationed by off-line reversed phase-high-performance liquid chromatography, the peptides were analyzed by a TripleTOF 5600 mass spectrometer (AB SCIEX) coupled online to an Eksigent nanoLC Ultra in Information Dependent Mode and with iTRAQ reagent collision energy adjustment on. Data are available via ProteomeXchange with identifier PXD033436.

In this work, we analyzed the liver proteome as one of the series of studies on the role of graded CR levels (4,6,10,29,48).

### Correlation, Enrichment, and Pathway Mapping Method

The intensity for protein expression of each gene was correlated with the increase in CR level (24AL, 12AL, 10%CR, 20%CR, 30%CR, 40%CR), which was transferred into a numeric vector (0, 0, 10, 20, 30, 40), by Pearson correlation method in the statistical environment R (version 4.1.1; https://www.R-project.org/) mainly using the Hmisc package and the for a loop. The adjusted *p* value of the correlation test was obtained using the Benjamini–Hochberg method. DAVID: Functional Annotation Tools (https://david.ncifcrf.gov/tools.jsp) was used for enrichment analyses, both based on G.O. and KEGG pathways (49,50), and KEGG Mapper—Color Pathway (https://www.kegg.jp/kegg/tool/map_pathway2.html) was used for pathway mapping (51).

We validated the top 30 genes correlated either negatively or positively and the 30 least correlated genes of the transcriptome (29) with the increase of CR against the proteome. The RNA expression levels of each gene for each individual mouse were correlated with their corresponding protein expression levels (Pearson correlation).

## Supplementary Material

glad017_suppl_Supplementary_Material
