# Supplementary material for: The Effects of Graded Levels of Calorie Restriction: XIX. Impact of Graded Calorie Restriction on Protein Expression in the Liver
Source: J Gerontol A Biol Sci Med Sci. 2023 Feb 9;78(7):1125–34. doi: 10.1093/gerona/glad017 (PMC10329235; doi:10.1093/gerona/glad017)
Supplement: glad017_suppl_Supplementary_Material [file glad017_suppl_supplementary_material.pdf]

Online-Only Supplemental eTables, eFigures and eMethods

The effects of graded levels of calorie restriction: XIX. Impact of graded calorie restriction on protein expression in the liver

Lu Wang (PhD) [1,*], Davina Derous (PhD) [2,*], Xiahe Huang (PhD) [3,*], Sharon E. Mitchell (PhD) [2,*], Alex Douglas (PhD) [2], David Lusseau (PhD) [2], Yingchun Wang (PhD) [3,#] and John R. Speakman (PhD) [5,2,3,4,#]

1. School of Pharmacy, Collaborative Innovation Center of Advanced Drug Delivery System and Biotech Drugs in Universities of Shandong, Key Laboratory of Molecular Pharmacology and Drug Evaluation (Yantai University), Ministry of Education, Yantai University, Yantai, 264005, China
2. Institute of Biological and Environmental Sciences, University of Aberdeen, Aberdeen, Scotland, UK
3. State Key laboratory of Molecular Developmental Biology, Institute of Genetics and Developmental Biology, Chinese Academy of Sciences, Chaoyang, Beijing, P.R.China 100101
4. CAS Centre for Excellence in Animal Evolution and Genetics (CCEAEG), Kunming, China
5. Shenzhen key laboratory of metabolic health, Center for Energy Metabolism and Reproduction, Shenzhen Institutes of Advanced technology, Chinese Academy of Sciences, Shenzhen, China

*Equal contribution

[#]Correspondence to:

JRS: Email: j.speakman@abdn.ac.uk (lead contact)
Tel: +44 (0) 1224 272879 cell: +86 158 108 68669
Fax: +44 (0) 1224 272396

YCW: Email: ycwang@genetics.ac.cn
Tel: + 86 (0) 10 64806149

eTable 1. Significant correlations* between protein expressions of genes in metabolic pathways and the increase in calorie restriction (CR) level.

Symbols	Gene names	Correlation coefficients	*P* values	Pathways
Cbr1	carbonyl reductase 1	0.7482	0.0000	Arachidonic acid metabolism
Ephx2	epoxide hydrolase 2, cytoplasmic	-0.3246	0.0244	Arachidonic acid metabolism
Arg1	arginase, liver	0.5830	0.0000	Arginine biosynthesis
Asl	argininosuccinate lyase	0.5589	0.0000	Arginine biosynthesis
Ass1	argininosuccinate synthetase 1	0.5329	0.0001	Arginine biosynthesis
Got1	glutamic-oxaloacetic transaminase 1, soluble	0.4261	0.0025	Arginine biosynthesis
Idh1	isocitrate dehydrogenase 1 (NADP+), soluble	0.4124	0.0036	Citrate cycle (TCA cycle)
Idh2	isocitrate dehydrogenase 2 (NADP+), mitochondrial	0.4179	0.0031	Citrate cycle (TCA cycle)
Mdh2	malate dehydrogenase 2, NAD (mitochondrial)	0.4029	0.0045	Citrate cycle (TCA cycle)
Ahcy	S-adenosylhomocysteine hydrolase	0.4977	0.0003	Cysteine and methionine metabolism
Kyat3	kynurenine aminotransferase 3	0.6888	0.0000	Cysteine and methionine metabolism
Mat1a	methionine adenosyltransferase I, alpha	0.5882	0.0000	Cysteine and methionine metabolism
Acadl	acyl-Coenzyme A dehydrogenase, long-chain	0.4084	0.0040	Fatty acid degradation
Acadm	acyl-Coenzyme A dehydrogenase, medium chain	0.4128	0.0035	Fatty acid degradation
Acadvl	acyl-Coenzyme A dehydrogenase, very long chain	0.4607	0.0010	Fatty acid degradation
Acsl5	acyl-CoA synthetase long-chain family member 5	0.3183	0.0275	Fatty acid degradation
Cyp4a10	cytochrome P450, family 4, subfamily a, polypeptide 10	0.3280	0.0228	Fatty acid degradation

Symbols	Gene names	Correlation coefficients	*P* values	Pathways
Cyp4a14	cytochrome P450, family 4, subfamily a, polypeptide 14	0.7574	0.0000	Fatty acid degradation
Ehhadh	enoyl-Coenzyme A, hydratase/3-hydroxyacyl Coenzyme A dehydrogenase	0.6015	0.0000	Fatty acid degradation
Hadh	hydroxyacyl-Coenzyme A dehydrogenase	0.4114	0.0037	Fatty acid degradation
Cyp4a12a	cytochrome P450, family 4, subfamily a, polypeptide 12a	-0.4710	0.0037	Fatty acid degradation
Acot3	acyl-CoA thioesterase 3	0.4616	0.0010	Fatty acid elongation
Khk	ketohexokinase	0.3089	0.0326	Fructose and mannose metabolism
Tkfc	triokinase, FMN cyclase	0.4313	0.0022	Fructose and mannose metabolism
Cth	cystathionase (cystathionine gamma-lyase)	0.3584	0.0124	Glycine, serine and threonine metabolism
Maob	monoamine oxidase B	0.3993	0.0049	Glycine, serine and threonine metabolism
Sds	serine dehydratase	0.2953	0.0416	Glycine, serine and threonine metabolism
Shmt1	serine hydroxymethyltransferase 1 (soluble)	0.3272	0.0232	Glycine, serine and threonine metabolism
Bhmt	betaine-homocysteine methyltransferase	-0.4082	0.0040	Glycine, serine and threonine metabolism
Adh1	alcohol dehydrogenase 1 (class I)	0.4117	0.0036	Glycolysis / Gluconeogenesis
Adh5	alcohol dehydrogenase 5 (class III), chi polypeptide	0.2874	0.0476	Glycolysis / Gluconeogenesis
Aldh2	aldehyde dehydrogenase 2, mitochondrial	0.3049	0.0351	Glycolysis / Gluconeogenesis
Aldh3a2	aldehyde dehydrogenase family 3, subfamily A2	0.3213	0.0259	Glycolysis / Gluconeogenesis
Aldh9a1	aldehyde dehydrogenase 9, subfamily A1	0.4834	0.0005	Glycolysis / Gluconeogenesis
Aldob	aldolase B, fructose-bisphosphate	0.3721	0.0092	Glycolysis / Gluconeogenesis

Symbols	**Gene names**	**Correlation coefficients**	***P* values**	**Pathways**
Aldoc	aldolase C, fructose-bisphosphate	0.4716	0.0200	Glycolysis / Gluconeogenesis
Eno1	enolase 1, alpha non-neuron	0.5353	0.0001	Glycolysis / Gluconeogenesis
Gpi1	glucose-6-phosphate isomerase 1	0.6189	0.0000	Glycolysis / Gluconeogenesis
Pck1	phosphoenolpyruvate carboxykinase 1, cytosolic	0.2888	0.0465	Glycolysis / Gluconeogenesis
Pgk1	phosphoglycerate kinase 1	0.3600	0.0120	Glycolysis / Gluconeogenesis
Pgm2	phosphoglucomutase 2	0.3202	0.0265	Glycolysis / Gluconeogenesis
Tpi1	triosephosphate isomerase 1	0.4592	0.0010	Glycolysis / Gluconeogenesis
Hal	histidine ammonia lyase	0.2881	0.0470	Histidine metabolism
Uroc1	urocanase domain containing 1	0.3412	0.0176	Histidine metabolism
Aass	aminoadipate-semialdehyde synthase	0.3743	0.0088	Lysine degradation
Acot1	acyl-CoA thioesterase 1	0.6935	0.0000	Lysine degradation
Gsta4	glutathione S-transferase, alpha 4	0.5039	0.0003	Metabolism of xenobiotics by cytochrome P450
Hsd11b1	hydroxysteroid 11-beta dehydrogenase 1	0.5088	0.0002	Metabolism of xenobiotics by cytochrome P450
Gstm1	glutathione S-transferase, mu 1	0.6155	0.0000	Metabolism of xenobiotics by cytochrome P451
Gstt1	glutathione S-transferase, theta 1	0.4849	0.0005	Metabolism of xenobiotics by cytochrome P453
Gstt3	glutathione S-transferase, theta 3	0.4161	0.0033	Metabolism of xenobiotics by cytochrome P454
Mgst1	microsomal glutathione S-transferase 1	-0.4666	0.0008	Metabolism of xenobiotics by cytochrome P450

Symbols	Gene names	Correlation coefficients	*P* values	Pathways
Gstp1	glutathione S-transferase, pi 1	-0.6528	0.0000	Metabolism of xenobiotics by cytochrome P452
Car3	carbonic anhydrase 3	-0.5962	0.0000	Nitrogen metabolism
Ugt1a1	UDP glucuronosyltransferase 1 family, polypeptide A1	0.4009	0.0047	Pentose and glucuronate interconversions
Ugt1a6b	UDP glucuronosyltransferase 1 family, polypeptide A6B	0.3812	0.0075	Pentose and glucuronate interconversions
Ugt2b1	UDP glucuronosyltransferase 2 family, polypeptide B1	-0.4374	0.0019	Pentose and glucuronate interconversions
Tkt	transketolase	0.6719	0.0000	Pentose phosphate pathway
Keg1	kidney expressed gene 1	-0.5463	0.0001	Phenylalanine metabolism
Slc27a5	solute carrier family 27 (fatty acid transporter), member 5	0.3191	0.0270	Primary bile acid biosynthesis
Scp2	sterol carrier protein 2, liver	-0.4920	0.0004	Primary bile acid biosynthesis
Adk	adenosine kinase	0.3226	0.0253	Purine metabolism
Dpys	dihydropyrimidinase	0.3500	0.0147	Pyrimidine metabolism
Upb1	ureidopropionase, beta	0.4031	0.0081	Pyrimidine metabolism
Me1	malic enzyme 1, NADP(+)-dependent, cytosolic	0.3015	0.0373	Pyruvate metabolism
Rdh9	retinol dehydrogenase 9	0.4311	0.0022	Retinol metabolism
Blvrb	biliverdin reductase B (flavin reductase (NADPH))	0.4453	0.0015	Riboflavin metabolism
Cyp2b9	cytochrome P450, family 2, subfamily b, polypeptide 9	0.3690	0.0268	Steroid hormone biosynthesis
Cyp2c68	cytochrome P450, family 2, subfamily c, polypeptide 68	0.3277	0.0341	Steroid hormone biosynthesis

**Symbols**	**Gene names**	**Correlation coefficients**	***P* values**	**Pathways**
Hsd3b5	hydroxy-delta-5-steroid dehydrogenase, 3 beta- and steroid delta-isomerase 5	-0.3722	0.0092	Steroid hormone biosynthesis
Comt	catechol-O-methyltransferase	-0.3877	0.0065	Steroid hormone biosynthesis
Selenbp2	selenium binding protein 2	-0.5243	0.0010	Sulfur metabolism
Csad	cysteine sulfinic acid decarboxylase	0.3661	0.0105	Taurine and hypotaurine metabolism
Kynu	kynureninase	-0.3061	0.0344	Tryptophan metabolism
Hpd	4-hydroxyphenylpyruvic acid dioxygenase	0.4218	0.0028	Tyrosine metabolism
Hmgcs2	3-hydroxy-3-methylglutaryl-Coenzyme A synthase 2	0.4947	0.0004	Valine, leucine and isoleucine degradation
Aox3	aldehyde oxidase 3	-0.4697	0.0008	Valine, leucine and isoleucine degradation

Note: the metabolic pathways is Kyoto Encyclopedia of Genes and Genomes (KEGG) global and overview map of metabolism.

*: by Pearson correlation method.

eTable 2. Correlations* between protein or mRNA expression of genes in insulin/IGF-1, NF-kB, mTOR and sirtuins pathways and the increase of calorie restriction (CR), and between the protein and the mRNA expression.

Symbols	Gene names	Protein expression vs increased CR level		mRNA expression vs increased CR level		Protein vs mRNA expression	
		Correlation coefficients	*P* values	Correlation coefficients	*P* values	Correlation coefficients	*P* values
Cat	catalase	-0.1631	0.2681	-0.7473	$<10^{-7}$	0.006417	0.9691
Csnk2b	casein kinase 2, beta polypeptide	0.04388	0.8628	-0.3452	0.02708	0.03685	0.9005
Deptor	DEP domain containing MTOR-interacting protein	0.01715	0.9209	-0.3287	0.03586	0.04804	0.8046
Eif4e	eukaryotic translation initiation factor 4E	0.08135	0.6085	-0.3740	0.01510	-0.00815	0.9624
Grb2	growth factor receptor bound protein 2	-0.1948	0.4386	-0.3854	0.01282	0.1612	0.5365
Insr	insulin receptor	0.2289	0.3459	0.3763	0.01531	-0.3498	0.1548
Map2k1	mitogen-activated protein kinase kinase 1	0.02487	0.8855	-0.5422	0.00025	0.2780	0.1299
Parp1	poly (ADP-ribose) polymerase family, member 1	-0.011	0.9655	-0.5355	0.000309	0.2490	0.3708
Ppargc1a	peroxisome proliferative activated receptor, gamma, coactivator 1 alpha	0.1263	0.6176	0.7280	$<10^{-7}$	0.1009	0.7429
Prkag1	protein kinase, AMP-activated, gamma 1 non-catalytic subunit	0.1515	0.4243	0.6387	$<10^{-5}$	0.03543	0.8607
Sod2	superoxide dismutase 2, mitochondrial	0.1530	0.2991	0.5403	0.000266	0.02547	0.8777
Akt1s1	AKT1 substrate 1 (proline-rich)	0.2095	0.5135	-0.3909	0.01151	-0.2553	0.4486
Atp6v1h	ATPase, H+ transporting, lysosomal V1 subunit H	0.03682	0.9095	-0.3167	0.04365	0.2089	0.5375
Eif4ebp1	eukaryotic translation initiation factor 4E binding protein 1	0.2311	0.6595	-0.3635	0.01950	-0.07483	0.9048
Clip1	CAP-GLY domain containing linker protein 1	-0.09424	0.8802	0.7087	$<10^{-6}$	0.7376	0.1549

Symbols	Gene names	Protein expression vs increased CR level		mRNA expression vs increased CR level		Protein vs mRNA expression	
		**Correlation coefficients**	***P* values**	**Correlation coefficients**	***P* values**	**Correlation coefficients**	***P* values**
Atp6v1g2	ATPase, H+ transporting, lysosomal V1 subunit G2	-0.7312	0.09870	-0.3418	0.02874	-0.6923	0.5132
Akt1s1	AKT1 substrate 1 (proline-rich)	0.2095	0.5135	-0.3909	0.01151	-0.2553	0.4486
Lyn	Yamaguchi sarcoma viral (v-yes-1) oncogene homolog	0.1419	0.6600	-0.4522	0.002997	-0.2416	0.5312
Rela	v-rel reticuloendotheliosis viral oncogene homolog A (avian)	0.2316	0.5488	-0.4534	0.002908	0.5573	0.2506

Note: the insulin/IGF-1, NF-kB, mTOR and sirtuins pathways were obtained from Kyoto Encyclopedia of Genes and Genomes (KEGG).

*: by Pearson correlation method.

eFigure 1. Principal Component Analysis (PCA) for the protein expression in liver of the in total 2206 genes from the individuals in each calorie restriction (CR) group.

24AL stands for mice being fed *ad libitum* for 24h a day (n=8); 12AL stands for mice being fed *ad libitum* for 12h a day during the dark period (n=8); 10%CR (n=8), 20%CR (n=8), 30%CR (n=8) and 40%CR (n=8) indicates mice being fed 10%, 20%, 30% and 40% lower calories respectively than their own individual intakes measured over a baseline period of 14 days prior to introducing CR.

eFigure 2. Enrichment analysis based on G.O. for the 134 genes with protein expression significantly (*P* < 0.05) correlated to the increase in calorie restriction (CR).

Y-axis: twenty-seven G.O. terms with Benjamini-adjusted *P* values < 0.05 and enriched by more than 10 (included) genes; X-axis: Benjamini-adjusted *P* values; color from green to orange: $-\log_{10}$(Benjamini-adjusted *P* values) from small to big; size of dot: the count of enriched genes.

eFigure 3. Enrichment analysis based on Kyoto Encyclopedia of Genes and Genomes (KEGG) pathways for the 134 genes with protein expression significantly (*P* < 0.05) correlated to the increase in calorie restriction (CR).

Y-axis: thirty-three KEGG pathways with Benjamini-adjusted *P* values < 0.05; X-axis: Benjamini-adjusted *P* values; color from green to purple: $-\log_{10}$(Benjamini-adjusted *P* values) from small to big; size of dot: the count of enriched proteins.

eFigure 4. Protein expression of genes in Glycolysis pathway that were significantly correlated (*P*< 0.05) with the increase of CR plotted against the calorie restriction (CR) levels.

The protein expression of genes were correlated with the increase in CR level by Pearson correlation method. The X-axis represents the CR level and the Y-axis represents the intensity value of protein expression. 24AL stands for mice being fed *ad libitum* for 24h a day (n=8); 12AL stands for mice being fed *ad libitum* for 12h a day during the dark period (n=8); 10%CR (n=8), 20%CR (n=8), 30%CR (n=8) and 40%CR (n=8) indicates mice being fed 10%, 20%, 30% and 40% lower calories respectively than their own individual intakes measured over a baseline period of 14 days prior to introducing CR. The pathway was obtained from Kyoto Encyclopedia of Genes and Genomes (KEGG).

eFigure 5. Citrate cycle (TCA cycle) diagram showing Pearson correlation of protein expression of the mapped genes with the increased calorie restriction (CR) level.

The pathway was colored based on the correlation coefficients and *P* values, red standing for significant (*P*< 0.05) positive correlation; pink positive correlation with *P*>= 0.05; blue significant (*P*< 0.05) negative correlation; cyan negative correlation with *P*>= 0.05. EC: 4.1.1.32 = phosphoenolpyruvate carboxykinase 1, cytosolic (Pck1); EC: 1.1.1.37 = malate

dehydrogenase 2, NAD (mitochondrial) (Mdh2); EC: 1.1.1.42 = isocitrate dehydrogenase 1 (NADP+), soluble (Idh1) / isocitrate dehydrogenase 2 (NADP+), mitochondrial (Idh2). The pathway was obtained from Kyoto Encyclopedia of Genes and Genomes (KEGG).

eFigure 6. Pentose phosphate pathway diagram showing Pearson correlation of protein expression of the mapped genes with the increased calorie restriction (CR) level.

The pathway was colored based on the correlation coefficients and *P* values, red standing for significant (*P*< 0.05) positive correlation; pink positive correlation with *P*>= 0.05; blue significant (*P*< 0.05) negative correlation; cyan negative correlation with *P*>= 0.05. EC: 5.3.1.9 = glucose-6-phosphate isomerase 1 (Gpi1); EC: 4.1.2.13 = aldolase B, fructose-bisphosphate (Aldob) / aldolase C, fructose-bisphosphate (Aldoc); EC: 2.2.1.1 = transketolase (Tkt); EC: 5.4.2.2 / 5.4.2.7 = phosphoglucomutase 2 (Pgm2). The pathway was obtained from Kyoto Encyclopedia of Genes and Genomes (KEGG).

eFigure 7. Glyoxylate and dicarboxylate metabolism pathway diagram showing Pearson correlation of protein expression of the mapped genes with the increased calorie restriction (CR) level.

The pathway was colored based on the correlation coefficients and *P* values, red standing for significant (*P*< 0.05) positive correlation; pink positive correlation with *P*>= 0.05; blue significant (*P*< 0.05) negative correlation; cyan negative correlation with *P*>= 0.05. EC: 1.1.1.37 = malate dehydrogenase 2, NAD (mitochondrial) (Mdh2); EC: 2.1.2.1 = serine hydroxymethyltransferase 1 (soluble) (Shmt1). The pathway was obtained from Kyoto Encyclopedia of Genes and Genomes (KEGG).

eFigure 8. Protein expression of genes in Fatty acid degradation pathway that were significantly correlated (*P*< 0.05) with the increase of CR plotted against the calorie restriction (CR) levels.

The protein expression of genes were correlated with the increase in CR level by Pearson correlation method. The X-axis represents the CR level and the Y-axis represents the intensity value of protein expression. 24AL stands for mice being fed *ad libitum* for 24h a day (n=8); 12AL stands for mice being fed *ad libitum* for 12h a day during the dark period (n=8); 10%CR (n=8), 20%CR (n=8), 30%CR (n=8) and 40%CR (n=8) indicates mice being fed 10%, 20%, 30% and 40% lower calories respectively than their own individual intakes measured over a baseline period of 14 days prior to introducing CR. The pathway was obtained from Kyoto Encyclopedia of Genes and Genomes (KEGG).

eFigure 9. PPAR signaling pathway diagram showing Pearson correlation of protein expression of the mapped genes with the increased calorie restriction (CR) level.

The pathway was colored based on the correlation coefficients and *P* values, red standing for significant (*P*< 0.05) positive correlation; pink positive correlation with *P*>= 0.05; blue significant (*P*< 0.05) negative correlation; cyan negative correlation with *P*>= 0.05. The pathway was obtained from Kyoto Encyclopedia of Genes and Genomes (KEGG).

eFigure 10. Biosynthesis of amino acids pathways diagram showing Pearson correlation of protein expression of the mapped genes with the increased calorie restriction (CR) level.

The pathway was colored based on the correlation coefficients and *P* values, red standing for significant ($P< 0.05$) positive correlation; pink positive correlation with $P>= 0.05$; blue significant ($P< 0.05$) negative correlation; cyan negative correlation with $P>= 0.05$. The pathway was obtained from Kyoto Encyclopedia of Genes and Genomes (KEGG).

eFigure 11. Arginine biosynthesis pathways diagram showing Pearson correlation of protein expression of the mapped genes with the increased calorie restriction (CR) level.

The pathway was colored based on the correlation coefficients and *P* values, red standing for significant ($P< 0.05$) positive correlation; pink positive correlation with $P>= 0.05$; blue significant ($P< 0.05$) negative correlation; cyan negative correlation with $P>= 0.05$. EC: 4.3.2.1 = argininosuccinate lyase (Asl); EC: 3.5.3.1 = arginase, liver (Arg1); EC: 6.3.4.5 = argininosuccinate synthetase 1 (Ass1); EC: 2.6.1.1 = glutamic-oxaloacetic transaminase 1, also known as aspartate aminotransferase (Got1). The pathway was obtained from Kyoto Encyclopedia of Genes and Genomes (KEGG).

eFigure 12. Valine, leucine and isoleucine degradation pathway diagram showing Pearson correlation of protein expression of the mapped genes with the increased calorie restriction (CR) level.

The pathway was colored based on the correlation coefficients and *P* values, red standing for significant ($P< 0.05$) positive correlation; pink positive correlation with $P>= 0.05$; blue significant ($P< 0.05$) negative correlation; cyan negative correlation with $P>= 0.05$. EC: 1.3.8.7 = acyl-Coenzyme A dehydrogenase, medium chain (Acadm); EC: 4.2.1.17 = enoyl-Coenzyme A, hydratase/3-hydroxyacyl Coenzyme A dehydrogenase (Ehhadh); EC: 1.1.1.35 = hydroxyacyl-Coenzyme A dehydrogenase (Hadh); EC: 2.3.3.10 = 3-hydroxy-3-methylglutaryl-Coenzyme A synthase 2 (Hmgcs2); EC: 1.2.1.3 = aldehyde dehydrogenase (NAD+) (Aldh2, Aldh3a2 and Aldh9a1); EC: 1.2.3.1 = aldehyde oxidase 3 (Aox3). The pathway was obtained from Kyoto Encyclopedia of Genes and Genomes (KEGG).

eFigure 13. Lysine degradation pathway diagram showing Pearson correlation of protein expression of the mapped genes with the increased calorie restriction (CR) level.

The pathway was colored based on the correlation coefficients and *P* values, red standing for significant ($P< 0.05$) positive correlation; pink positive correlation with $P>= 0.05$; blue significant ($P< 0.05$) negative correlation; cyan negative correlation with $P>= 0.05$. EC: 1.5.1.8 / 1.5.1.9 = aminoadipate-semialdehyde synthase (Aass); EC: 4.2.1.17 = enoyl-Coenzyme A, hydratase/3-hydroxyacyl Coenzyme A dehydrogenase (Ehhadh); EC: 1.1.1.35 = hydroxyacyl-Coenzyme A dehydrogenase (Hadh); EC: 1.2.1.3 = aldehyde dehydrogenase (NAD+) (Aldh2, Aldh3a2 and Aldh9a1); EC: 1.2.1.47 = aldehyde dehydrogenase family 9 member A1

(Aldh9a1). The pathway was obtained from Kyoto Encyclopedia of Genes and Genomes (KEGG).

eFigure 14. Protein expression of genes in Metabolism pathway of xenobiotics by cytochrome P450 that were significantly correlated (*P*< 0.05) with the increase of CR plotted against the calorie restriction (CR) levels.

The protein expression of genes were correlated with the increase in CR level by Pearson correlation method. The X-axis represents the CR level and the Y-axis represents the intensity value of protein expression. 24AL stands for mice being fed *ad libitum* for 24h a day (n=8); 12AL stands for mice being fed *ad libitum* for 12h a day during the dark period (n=8); 10%CR (n=8), 20%CR (n=8), 30%CR (n=8) and 40%CR (n=8) indicates mice being fed 10%, 20%, 30% and 40% lower calories respectively than their own individual intakes measured over a baseline period of 14 days prior to introducing CR. The pathway was obtained from Kyoto Encyclopedia of Genes and Genomes (KEGG).

eFigure 15. Longevity regulating pathways diagram showing Pearson correlation of protein expression of the mapped genes with the increased calorie restriction (CR) level.

The longevity regulating pathways consist of insulin/ IGF-1 pathway, the SIRT pathway, the AMPK pathway and the mTOR signaling pathway. The pathways were colored based on the correlation coefficients and *P* values, red standing for significant (*P*< 0.05) positive correlation; pink positive correlation with *P*>= 0.05; blue significant (*P*< 0.05) negative correlation; cyan negative correlation with *P*>= 0.05. The pathway was obtained from Kyoto Encyclopedia of Genes and Genomes (KEGG).

eFigure 16. Protein expression of genes in Longevity regulating pathways plotted against the calorie restriction (CR) levels.

The X-axis represents the CR level and the Y-axis represents the intensity value of protein expression. 24AL stands for mice being fed *ad libitum* for 24h a day (n=8); 12AL stands for mice being fed *ad libitum* for 12h a day during the dark period (n=8); 10%CR (n=8), 20%CR (n=8), 30%CR (n=8) and 40%CR (n=8) indicates mice being fed 10%, 20%, 30% and 40% lower calories respectively than their own individual intakes measured over a baseline period of 14 days prior to introducing CR. The pathway was obtained from Kyoto Encyclopedia of Genes and Genomes (KEGG).

eFigure 17. mTOR signaling pathway diagram showing Pearson correlation of protein expression of the mapped genes with the increased calorie restriction (CR) level.

The pathway was colored based on the correlation coefficients and *P* values, red standing for significant (*P*< 0.05) positive correlation; pink positive correlation with *P*>= 0.05; blue significant (*P*< 0.05) negative correlation; cyan negative correlation with *P*>= 0.05. The pathway was obtained from Kyoto Encyclopedia of Genes and Genomes (KEGG).

eFigure 18. Protein expression of genes in mTOR signaling pathway plotted against the calorie restriction (CR) levels.

The X-axis represents the CR level and the Y-axis represents the intensity value of protein expression. 24AL stands for mice being fed *ad libitum* for 24h a day (n=8); 12AL stands for mice being fed *ad libitum* for 12h a day during the dark period (n=8); 10%CR (n=8), 20%CR (n=8), 30%CR (n=8) and 40%CR (n=8) indicates mice being fed 10%, 20%, 30% and 40% lower calories respectively than their own individual intakes measured over a baseline period of 14 days prior to introducing CR. The pathway was obtained from Kyoto Encyclopedia of Genes and Genomes (KEGG).

eFigure 19. NF-kappa B signaling (NF-κB) pathway diagram showing Pearson correlation of protein expression of the mapped genes with the increased calorie restriction (CR) level.

The pathway was colored based on the correlation coefficients and *P* values, red standing for significant (P< 0.05) positive correlation; pink positive correlation with P>= 0.05; blue significant (P< 0.05) negative correlation; cyan negative correlation with P>= 0.05. The pathway was obtained from Kyoto Encyclopedia of Genes and Genomes (KEGG).

eFigure 20. Protein expression of genes in NF-kappa B signaling (NF-κB) pathway plotted against the calorie restriction (CR) levels.

The X-axis represents the CR level and the Y-axis represents the intensity value of protein expression. 24AL stands for mice being fed *ad libitum* for 24h a day (n=8); 12AL stands for mice being fed *ad libitum* for 12h a day during the dark period (n=8); 10%CR (n=8), 20%CR (n=8), 30%CR (n=8) and 40%CR (n=8) indicates mice being fed 10%, 20%, 30% and 40% lower calories respectively than their own individual intakes measured over a baseline period of 14 days prior to introducing CR. The pathway was obtained from Kyoto Encyclopedia of Genes and Genomes (KEGG).

eFigure 21. Pathways in cancer diagram showing Pearson correlation of protein expression of the mapped genes with the increased calorie restriction (CR) level.

The pathways were colored based on the correlation coefficients and *P* values, red standing for significant (P< 0.05) positive correlation; pink positive correlation with P>= 0.05; blue significant (P< 0.05) negative correlation; cyan negative correlation with P>= 0.05. The pathway was obtained from Kyoto Encyclopedia of Genes and Genomes (KEGG).

eFigure 22. Graphical representation of the transcript-protein validation performed for the 90 target genes.

30 genes that significantly correlated with the increase of calorie restriction (CR) (red), 30 genes that did not correlate with the increase in CR (grey) and 30 genes that correlated negatively with the increase in CR (blue) were correlated with their corresponding protein levels. A significant positive association (p-value < 0.05, adjusted for multiple testing) was found between the transcript levels and protein levels for 47 of the 60 gene-protein comparisons that changed their expression with CR (purple). 29 of the 30 gene-protein

comparisons that changed did not change their expression with CR were not significant (yellow). The thickness of the lines represent the number of genes indicated by the numbers next the lines.

eFigure 23. Distribution of the correlation coefficient of the mRNA and protein validation of the 90 target genes.

30 genes that significantly correlated with the increase of calorie restriction (CR) (red), 30 genes that did not correlate with the increase in CR (grey) and 30 genes that correlated negatively with the increase in CR (blue) were correlated with their corresponding protein levels.

eFigure 1

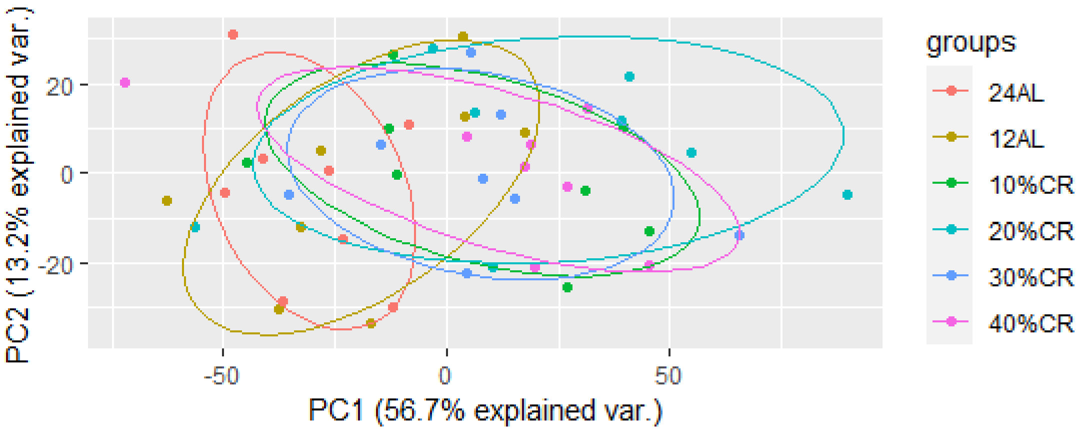

PC2 (13.2% explained var.)
20
0
-20
-50
0
50
PC1 (56.7% explained var.)
groups
24AL
12AL
10%CR
20%CR
30%CR
40%CR

eFigure 2

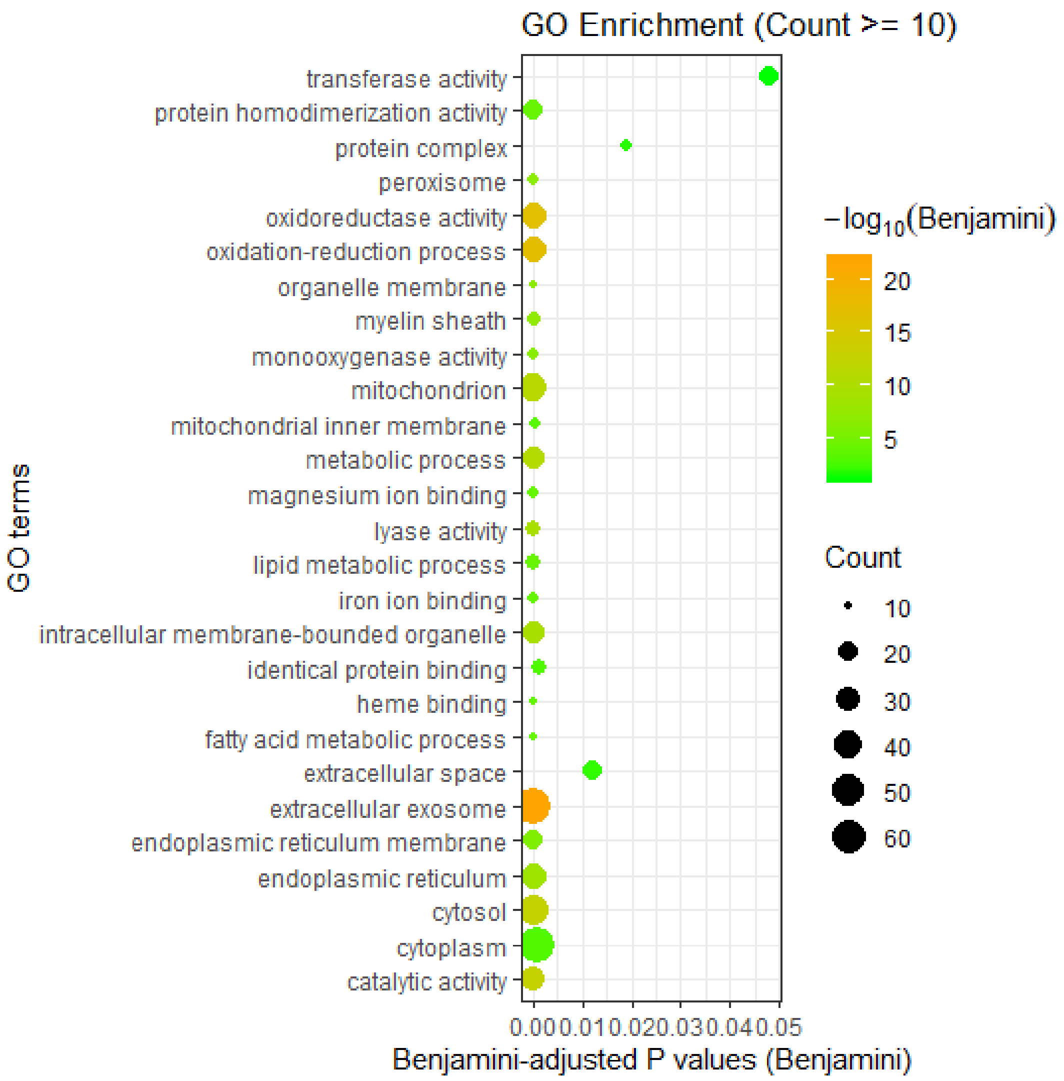

GO Enrichment (Count >= 10)
transferase activity
protein homodimerization activity
protein complex
peroxisome
oxidoreductase activity
oxidation-reduction process
organelle membrane
myelin sheath
monooxygenase activity
mitochondrion
mitochondrial inner membrane
metabolic process
magnesium ion binding
lyase activity
lipid metabolic process
iron ion binding
intracellular membrane-bounded organelle
identical protein binding
heme binding
fatty acid metabolic process
extracellular space
extracellular exosome
endoplasmic reticulum membrane
endoplasmic reticulum
cytosol
cytoplasm
catalytic activity
GO terms
0.00 0.01 0.02 0.03 0.04 0.05
Benjamini-adjusted P values (Benjamini)
−log10(Benjamini)
20
15
10
5
Count
10
20
30
40
50
60

eFigure 3

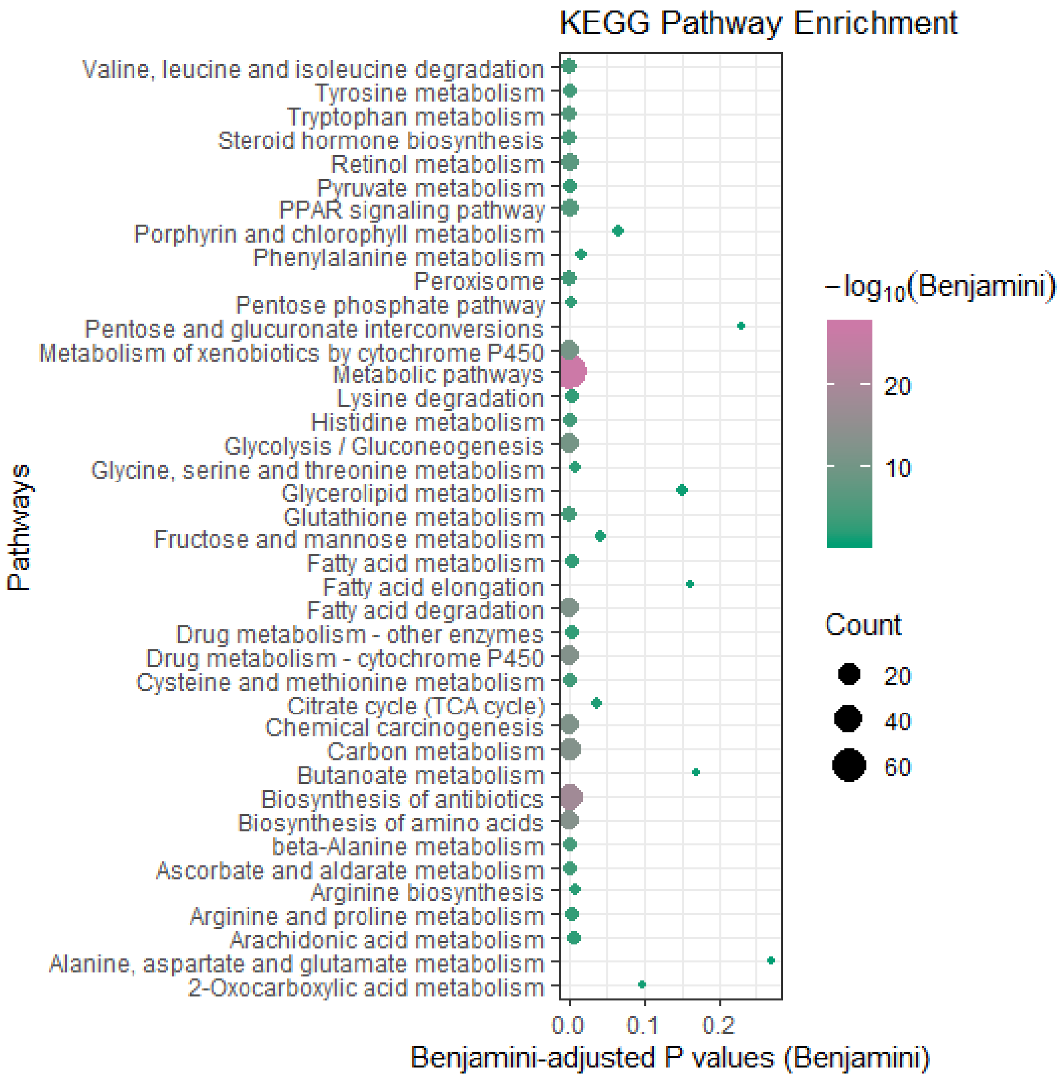
KEGG Pathway Enrichment
Valine, leucine and isoleucine degradation
Tyrosine metabolism
Tryptophan metabolism
Steroid hormone biosynthesis
Retinol metabolism
Pyruvate metabolism
PPAR signaling pathway
Porphyrin and chlorophyll metabolism
Phenylalanine metabolism
Peroxisome
Pentose phosphate pathway
Pentose and glucuronate interconversions
Metabolism of xenobiotics by cytochrome P450
Metabolic pathways
Lysine degradation
Histidine metabolism
Glycolysis / Gluconeogenesis
Glycine, serine and threonine metabolism
Glycerolipid metabolism
Glutathione metabolism
Fructose and mannose metabolism
Fatty acid metabolism
Fatty acid elongation
Fatty acid degradation
Drug metabolism - other enzymes
Drug metabolism - cytochrome P450
Cysteine and methionine metabolism
Citrate cycle (TCA cycle)
Chemical carcinogenesis
Carbon metabolism
Butanoate metabolism
Biosynthesis of antibiotics
Biosynthesis of amino acids
beta-Alanine metabolism
Ascorbate and aldarate metabolism
Arginine biosynthesis
Arginine and proline metabolism
Arachidonic acid metabolism
Alanine, aspartate and glutamate metabolism
2-Oxocarboxylic acid metabolism
Pathways
0.0
0.1
0.2
Benjamini-adjusted P values (Benjamini)
$-\log_{10}$(Benjamini)
20
10
Count
20
40
60

eFigure 4

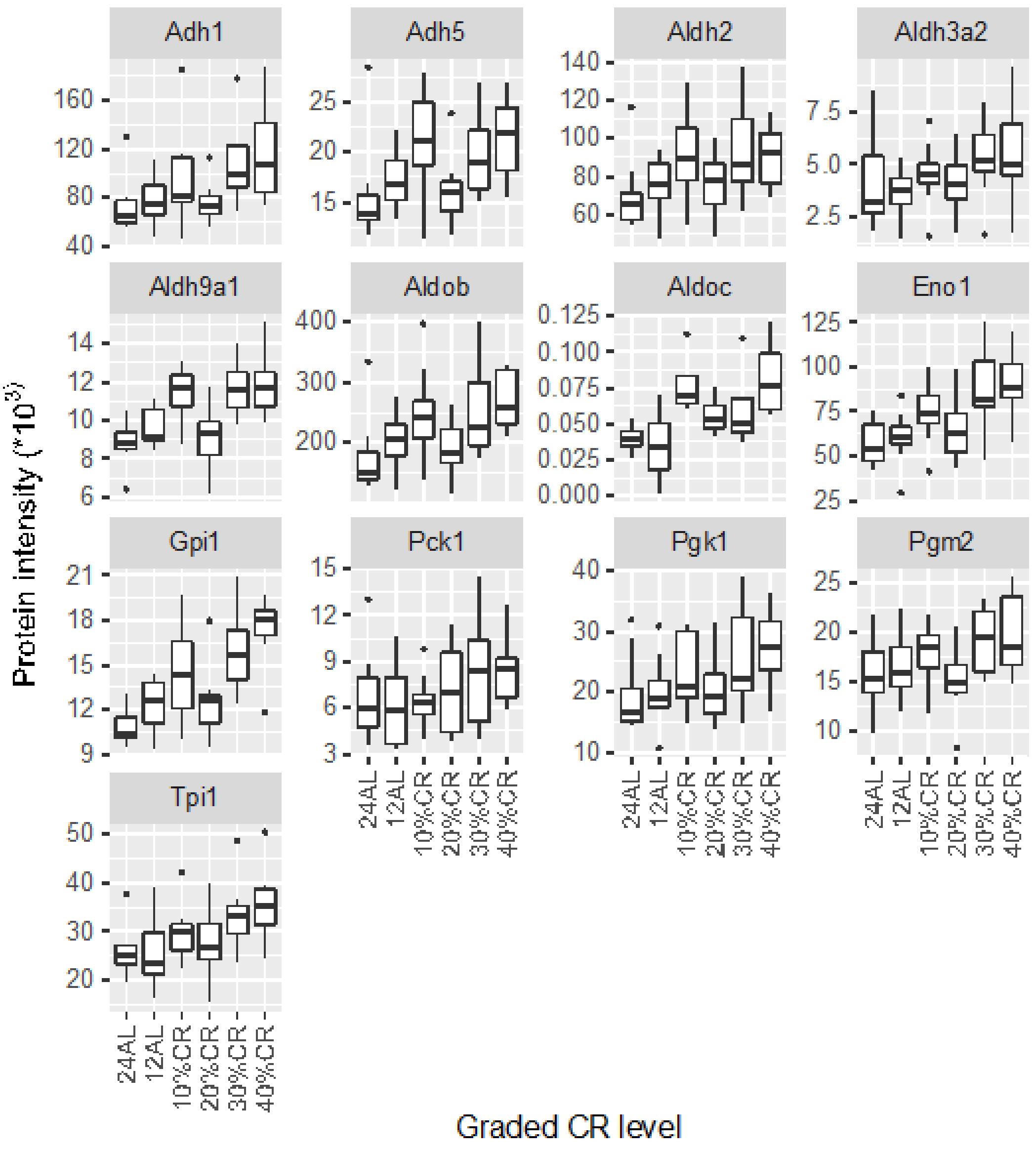
Adh1
Adh5
Aldh2
Aldh3a2
Aldh9a1
Aldob
Aldoc
Eno1
Gpi1
Pck1
Pgk1
Pgm2
Tpi1
Protein intensity (*10³)
24AL
12AL
10%CR
20%CR
30%CR
40%CR
Graded CR level

eFigure 5

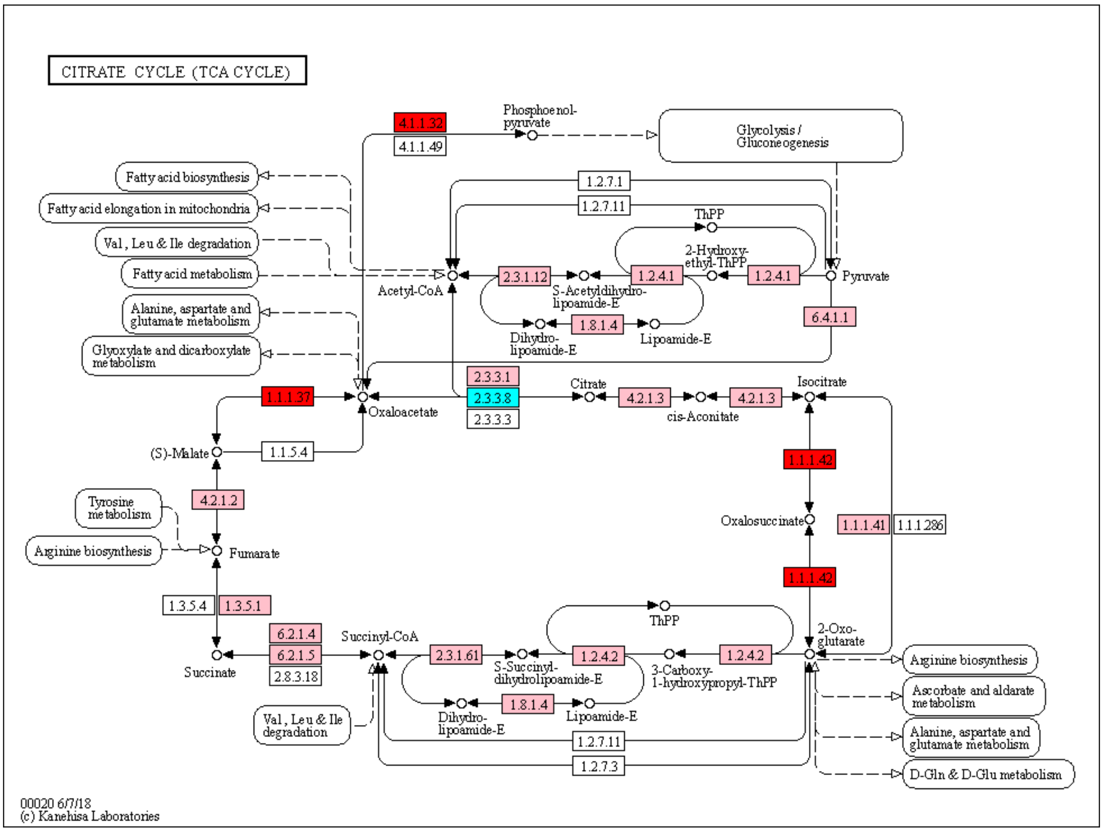

CITRATE CYCLE (TCA CYCLE)
4.1.1.32
4.1.1.49
Phosphoenol-pyruvate
Glycolysis / Gluconeogenesis
Fatty acid biosynthesis
Fatty acid elongation in mitochondria
Val, Leu & Ile degradation
Fatty acid metabolism
Alanine, aspartate and glutamate metabolism
Glyoxylate and dicarboxylate metabolism
1.2.7.1
1.2.7.11
ThPP
2-Hydroxy-ethyl-ThPP
Acetyl-CoA
2.3.1.12
S-Acetyldihydro-lipoamide-E
1.2.4.1
1.2.4.1
Pyruvate
1.8.1.4
Dihydro-lipoamide-E
Lipoamide-E
6.4.1.1
2.3.3.1
2.3.3.8
2.3.3.3
1.1.1.37
Oxaloacetate
Citrate
4.2.1.3
cis-Aconitate
4.2.1.3
Isocitrate
(S)-Malate
1.1.5.4
1.1.1.42
4.2.1.2
Tyrosine metabolism
Arginine biosynthesis
Fumarate
Oxalosuccinate
1.1.1.41
1.1.1.286
1.1.1.42
1.3.5.4
1.3.5.1
6.2.1.4
6.2.1.5
2.8.3.18
Succinate
Succinyl-CoA
2.3.1.61
S-Succinyl-dihydrolipoamide-E
1.2.4.2
3-Carboxy-1-hydroxypropyl-ThPP
1.2.4.2
2-Oxo-glutarate
Val, Leu & Ile degradation
1.8.1.4
Lipoamide-E
1.2.7.11
1.2.7.3
Arginine biosynthesis
Ascorbate and aldarate metabolism
Alanine, aspartate and glutamate metabolism
D-Gln & D-Glu metabolism
00020 6/7/18
(c) Kanehisa Laboratories

eFigure 6

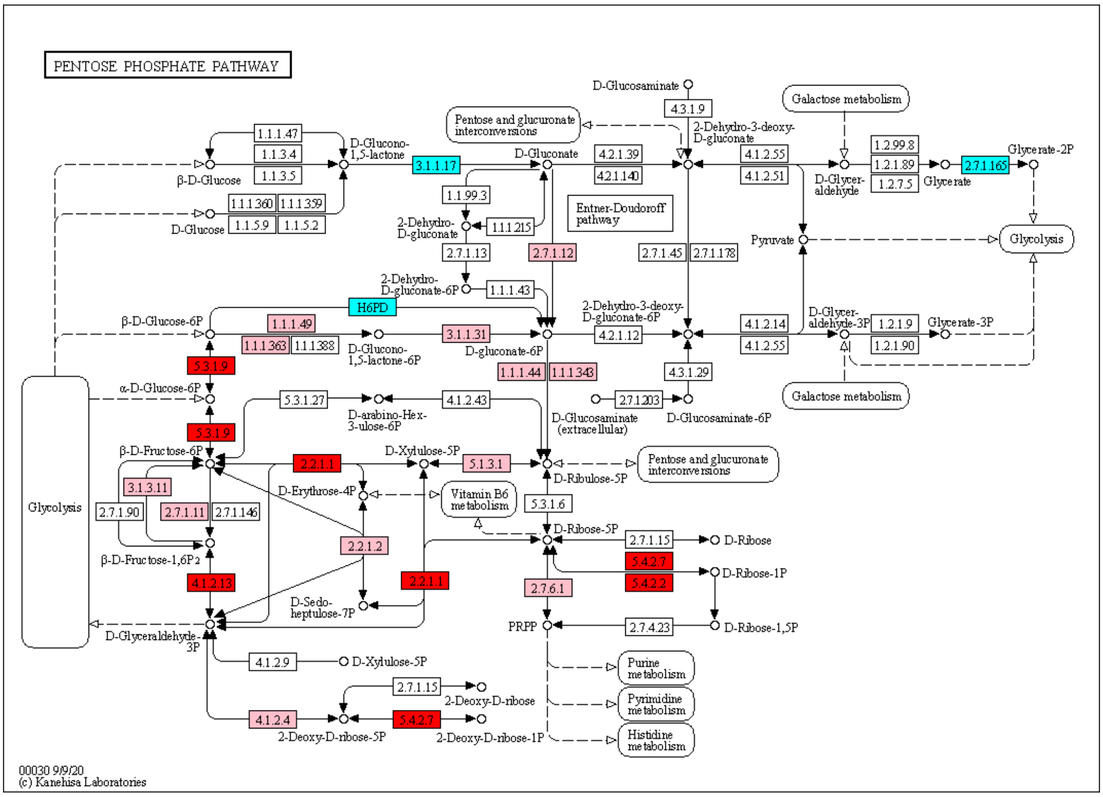
PENTOSE PHOSPHATE PATHWAY
D-Glucosaminate
4.3.1.9
Galactose metabolism
Pentose and glucuronate interconversions
2-Dehydro-3-deoxy-D-gluconate
1.1.1.47
1.1.3.4
1.1.3.5
D-Glucono-1,5-lactone
3.1.1.17
D-Gluconate
4.2.1.39
4.2.1.140
4.1.2.55
4.1.2.51
1.2.99.8
1.2.1.89
1.2.7.5
2.7.1.165
Glycerate-2P
Glycerate
D-Glyceraldehyde
β-D-Glucose
1.1.1.360
1.1.1.359
1.1.5.9
1.1.5.2
D-Glucose
1.1.99.3
2-Dehydro-D-gluconate
1.1.1.215
Entner-Doudoroff pathway
Pyruvate
Glycolysis
2.7.1.13
2.7.1.12
2.7.1.45
2.7.1.178
2-Dehydro-D-gluconate-6P
1.1.1.43
2-Dehydro-3-deoxy-D-gluconate-6P
β-D-Glucose-6P
H6PD
1.1.1.49
1.1.1.363
1.1.1.388
3.1.1.31
D-Glucono-1,5-lactone-6P
D-gluconate-6P
4.2.1.12
4.1.2.14
4.1.2.55
D-Glyceraldehyde-3P
1.2.1.9
1.2.1.90
Glycerate-3P
5.3.1.9
1.1.1.44
1.1.1.343
4.3.1.29
α-D-Glucose-6P
5.3.1.27
4.1.2.43
2.7.1.203
D-arabino-Hex-3-ulose-6P
D-Glucosaminate (extracellular)
D-Glucosaminate-6P
Galactose metabolism
5.3.1.9
β-D-Fructose-6P
2.2.1.1
D-Xylulose-5P
5.1.3.1
D-Ribulose-5P
Pentose and glucuronate interconversions
3.1.3.11
D-Erythrose-4P
Vitamin B6 metabolism
5.3.1.6
Glycolysis
2.7.1.90
2.7.1.11
2.7.1.146
D-Ribose-5P
2.7.1.15
D-Ribose
2.2.1.2
β-D-Fructose-1,6P2
5.4.2.7
5.4.2.2
D-Ribose-1P
4.1.2.13
2.2.1.1
2.7.6.1
D-Sedo-heptulose-7P
PRPP
2.7.4.23
D-Ribose-1,5P
D-Glyceraldehyde-3P
4.1.2.9
D-Xylulose-5P
Purine metabolism
2.7.1.15
2-Deoxy-D-ribose
Pyrimidine metabolism
4.1.2.4
5.4.2.7
2-Deoxy-D-ribose-5P
2-Deoxy-D-ribose-1P
Histidine metabolism
00030 9/9/20
(c) Kanehisa Laboratories

eFigure 7

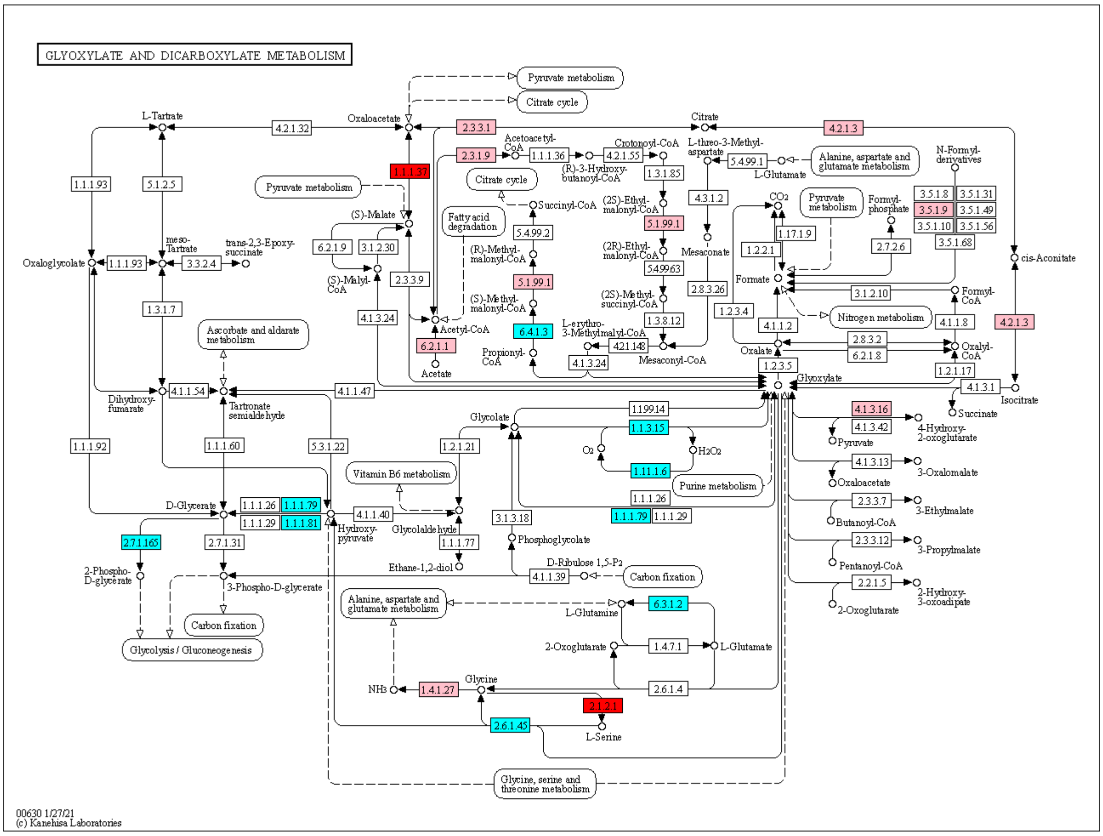
GLYOXYLATE AND DICARBOXYLATE METABOLISM
Pyruvate metabolism
Citrate cycle
L-Tartrate
Oxaloacetate
Citrate
4.2.1.32
2.3.3.1
4.2.1.3
1.1.1.37
2.3.1.9
Acetoacetyl-CoA
1.1.1.36
(R)-3-Hydroxy-butanoyl-CoA
Crotonoyl-CoA
4.2.1.55
1.3.1.85
L-threo-3-Methyl-aspartate
5.4.99.1
L-Glutamate
Alanine, aspartate and glutamate metabolism
N-Formyl-derivatives
1.1.1.93
5.1.2.5
Pyruvate metabolism
Citrate cycle
(S)-Malate
Fatty acid degradation
Succinyl-CoA
(2S)-Ethyl-malonyl-CoA
5.1.99.1
4.3.1.2
CO2
Pyruvate metabolism
Formyl-phosphate
3.5.1.8
3.5.1.31
3.5.1.9
3.5.1.49
3.5.1.10
3.5.1.56
3.5.1.68
Oxaloglycolate
1.1.1.93
meso-Tartrate
3.3.2.4
trans-2,3-Epoxy-succinate
6.2.1.9
3.1.2.30
(S)-Malyl-CoA
2.3.3.9
5.4.99.2
(R)-Methyl-malonyl-CoA
(2R)-Ethyl-malonyl-CoA
5.4.99.63
Mesaconate
1.17.1.9
1.2.1.2
2.7.2.6
cis-Aconitate
Formate
3.1.2.10
Formyl-CoA
1.3.1.7
5.1.99.1
(S)-Methyl-malonyl-CoA
(2S)-Methyl-succinyl-CoA
2.8.3.26
1.2.3.4
Nitrogen metabolism
4.1.1.8
4.2.1.3
4.1.3.24
Ascorbate and aldarate metabolism
Acetyl-CoA
6.4.1.3
L-erythro-3-Methylmalyl-CoA
1.3.8.12
4.1.1.2
2.8.3.2
Oxalyl-CoA
6.2.1.1
4.2.1.148
Oxalate
6.2.1.8
Propionyl-CoA
4.1.3.24
Mesaconyl-CoA
1.2.3.5
Acetate
1.2.1.17
Glyoxylate
4.1.3.1
Isocitrate
Dihydroxy-fumarate
4.1.1.54
Tartronate semialdehyde
4.1.1.47
1.1.99.14
4.1.3.16
Succinate
Glycolate
1.1.3.15
4.1.3.42
4-Hydroxy-2-oxoglutarate
Pyruvate
1.1.1.92
1.1.1.60
5.3.1.22
1.2.1.21
O2
H2O2
4.1.3.13
3-Oxalomalate
Vitamin B6 metabolism
1.11.1.6
Oxaloacetate
Purine metabolism
D-Glycerate
1.1.1.26
1.1.1.79
4.1.1.40
1.1.1.26
2.3.3.7
3-Ethylmalate
1.1.1.29
1.1.1.81
Hydroxy-pyruvate
Glycolaldehyde
3.1.3.18
1.1.1.79
1.1.1.29
Butanoyl-CoA
2.7.1.165
2.7.1.31
1.1.1.77
Phosphoglycolate
2.3.3.12
3-Propylmalate
2-Phospho-D-glycerate
Ethane-1,2-diol
D-Ribulose 1,5-P2
Pentanoyl-CoA
3-Phospho-D-glycerate
4.1.1.39
Carbon fixation
2.2.1.5
2-Hydroxy-3-oxoadipate
Alanine, aspartate and glutamate metabolism
L-Glutamine
6.3.1.2
2-Oxoglutarate
Carbon fixation
Glycolysis / Gluconeogenesis
2-Oxoglutarate
1.4.7.1
L-Glutamate
Glycine
NH3
1.4.1.27
2.6.1.4
2.1.2.1
2.6.1.45
L-Serine
Glycine, serine and threonine metabolism
00630 1/27/21
(c) Kanehisa Laboratories

eFigure 8

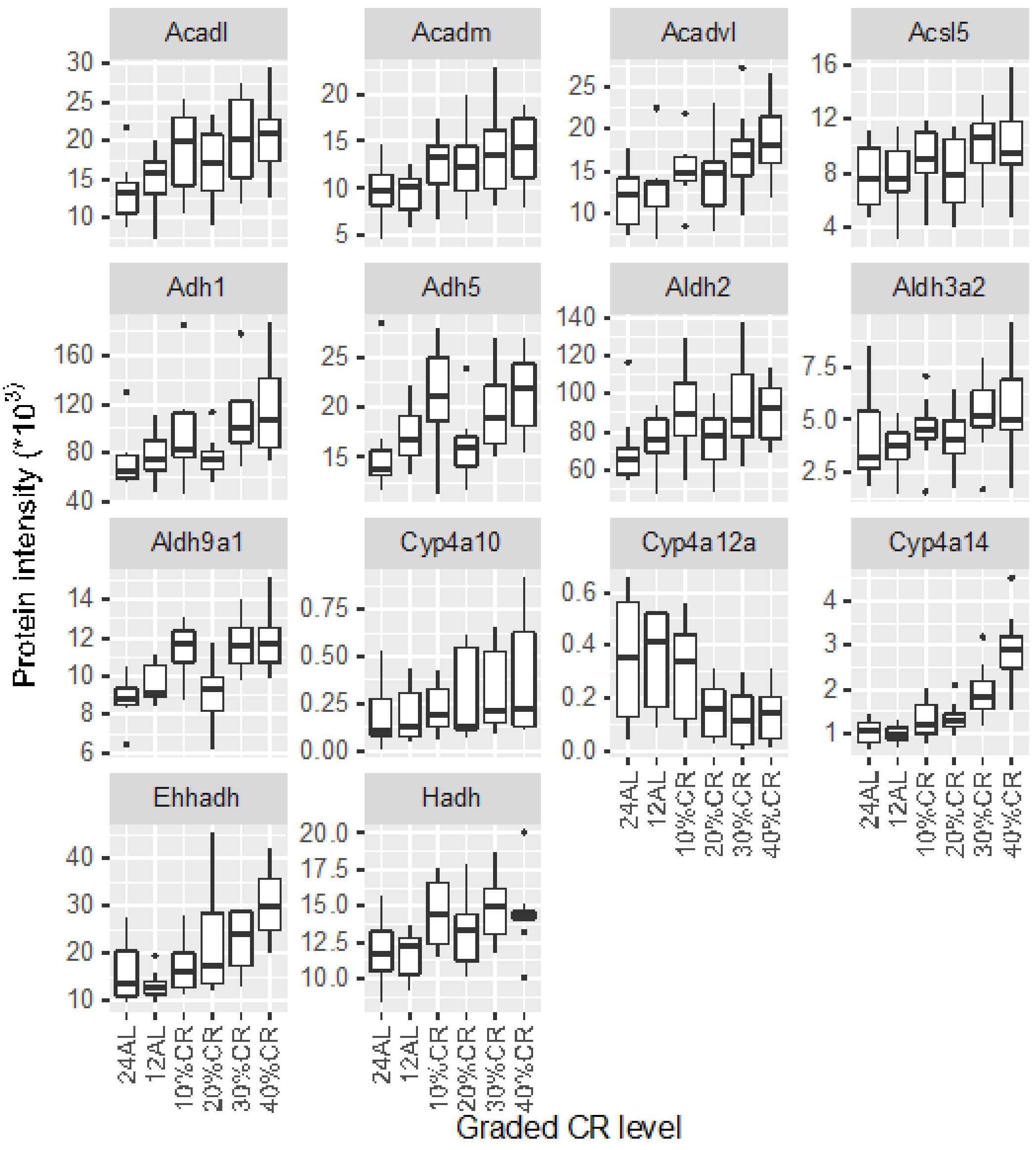

Acadl
Acadm
Acadvl
Acsl5
Adh1
Adh5
Aldh2
Aldh3a2
Aldh9a1
Cyp4a10
Cyp4a12a
Cyp4a14
Ehhadh
Hadh
Protein intensity (*10^3)
24AL
12AL
10%CR
20%CR
30%CR
40%CR
Graded CR level

eFigure 9

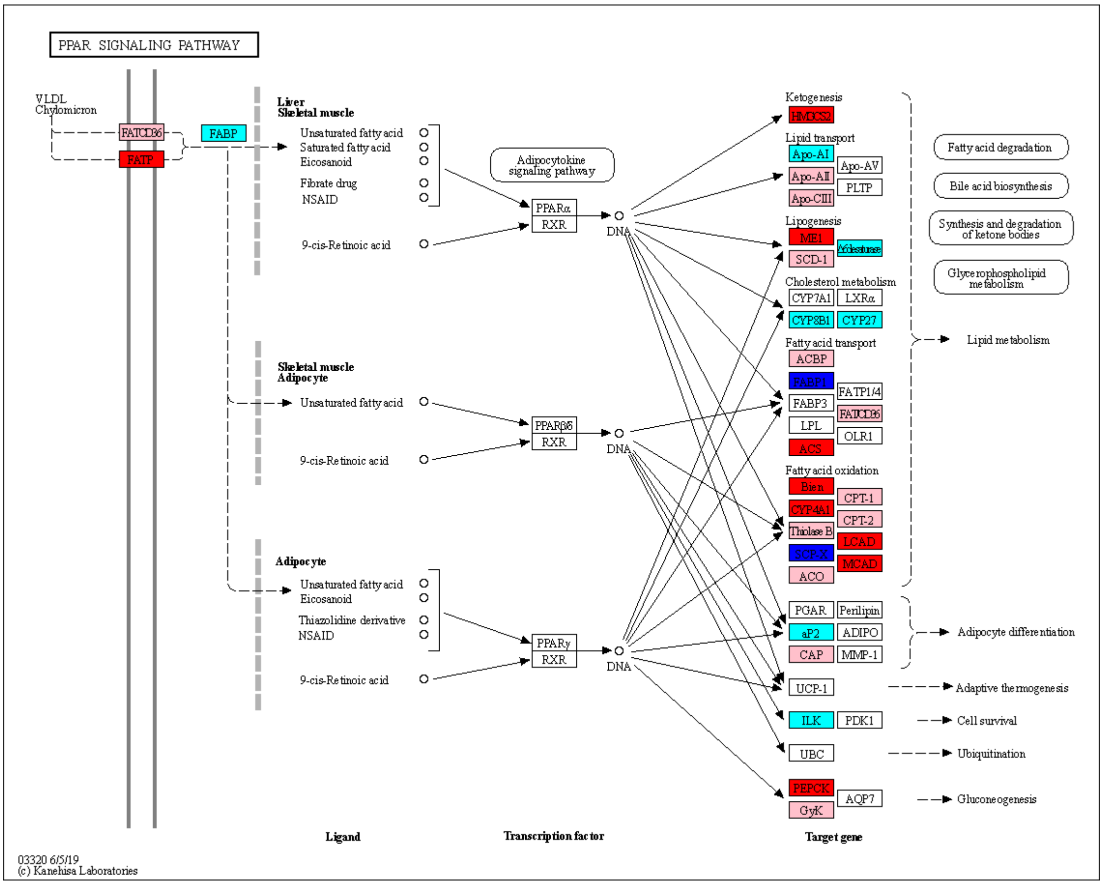
PPAR SIGNALING PATHWAY
VLDL
Chylomicron
FAT/CD36
FATP
FABP
Liver
Skeletal muscle
Unsaturated fatty acid
Saturated fatty acid
Eicosanoid
Fibrate drug
NSAID
9-cis-Retinoic acid
Adipocytokine signaling pathway
PPARα
RXR
DNA
Skeletal muscle
Adipocyte
PPARβ/δ
Thiazolidine derivative
PPARγ
Ketogenesis
HMGCS2
Lipid transport
Apo-AI
Apo-AII
Apo-AV
Apo-CIII
PLTP
Lipogenesis
ME1
Δ6-desaturase
SCD-1
Cholesterol metabolism
CYP7A1
LXRα
CYP8B1
CYP27
Fatty acid transport
ACBP
FABP1
FATP1/4
FABP3
LPL
OLR1
ACS
Fatty acid oxidation
Bien
CPT-1
CYP4A1
CPT-2
Thiolase B
LCAD
SCP-X
MCAD
ACO
PGAR
Perilipin
aP2
ADIPO
CAP
MMP-1
UCP-1
ILK
PDK1
UBC
PEPCK
AQP7
GyK
Fatty acid degradation
Bile acid biosynthesis
Synthesis and degradation of ketone bodies
Glycerophospholipid metabolism
Lipid metabolism
Adipocyte differentiation
Adaptive thermogenesis
Cell survival
Ubiquitination
Gluconeogenesis
Ligand
Transcription factor
Target gene
03320 6/5/19
(c) Kanehisa Laboratories

eFigure 10

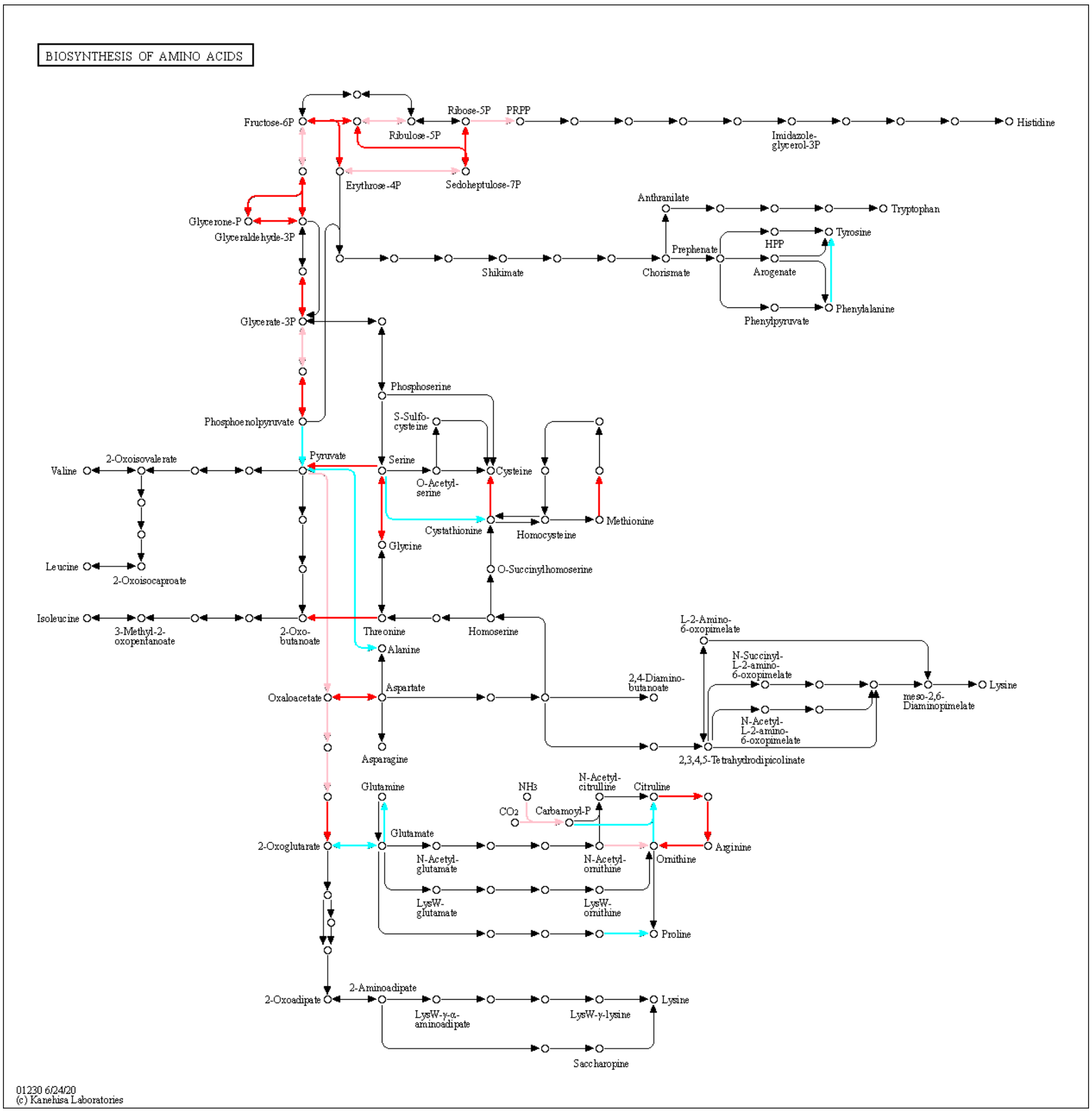
BIOSYNTHESIS OF AMINO ACIDS
Fructose-6P
Ribose-5P
PRPP
Ribulose-5P
Histidine
Imidazole-glycerol-3P
Erythrose-4P
Sedoheptulose-7P
Anthranilate
Tryptophan
Glycerone-P
Glyceraldehyde-3P
Tyrosine
HPP
Prephenate
Shikimate
Chorismate
Arogenate
Phenylalanine
Phenylpyruvate
Glycerate-3P
Phosphoserine
Phosphoenolpyruvate
S-Sulfo-cysteine
2-Oxoisovalerate
Pyruvate
Serine
Valine
Cysteine
O-Acetyl-serine
Methionine
Cystathionine
Homocysteine
Glycine
Leucine
2-Oxoisocaproate
O-Succinylhomoserine
Isoleucine
3-Methyl-2-oxopentanoate
2-Oxo-butanoate
Threonine
Homoserine
L-2-Amino-6-oxopimelate
Alanine
N-Succinyl-L-2-amino-6-oxopimelate
2,4-Diamino-butanoate
Aspartate
Lysine
Oxaloacetate
meso-2,6-Diaminopimelate
N-Acetyl-L-2-amino-6-oxopimelate
Asparagine
2,3,4,5-Tetrahydrodipicolinate
N-Acetyl-citrulline
Glutamine
NH3
Citruline
CO2
Carbamoyl-P
Glutamate
2-Oxoglutarate
Ornithine
Arginine
N-Acetyl-glutamate
N-Acetyl-ornithine
LysW-glutamate
LysW-ornithine
Proline
2-Aminoadipate
2-Oxoadipate
Lysine
LysW-γ-α-aminoadipate
LysW-γ-lysine
Saccharopine
01230 6/24/20
(c) Kanehisa Laboratories

eFigure 11

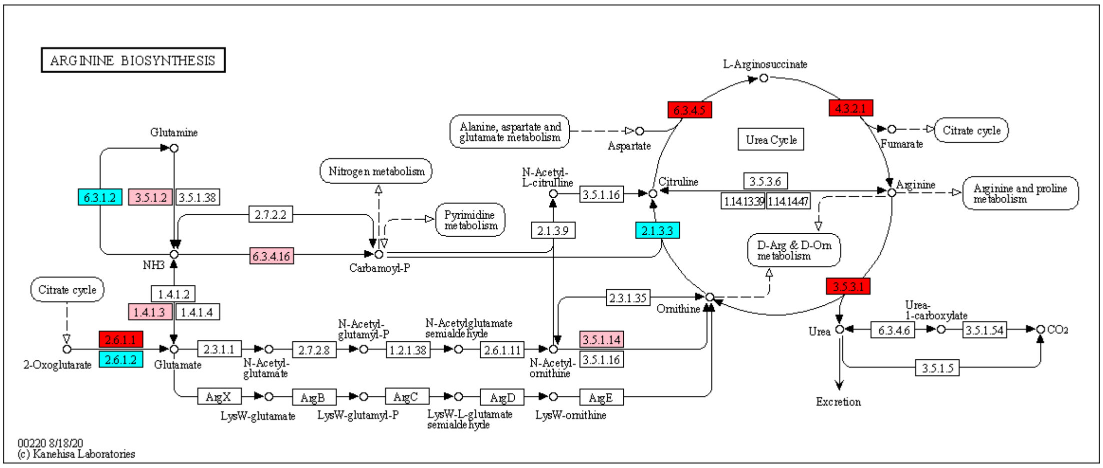
ARGININE BIOSYNTHESIS
Glutamine
6.3.1.2
3.5.1.2
3.5.1.38
2.7.2.2
NH3
6.3.4.16
Carbamoyl-P
Nitrogen metabolism
Pyrimidine metabolism
Alanine, aspartate and glutamate metabolism
Aspartate
L-Arginosuccinate
6.3.4.5
4.3.2.1
Fumarate
Citrate cycle
Urea Cycle
N-Acetyl-L-citrulline
3.5.1.16
Citrulline
3.5.3.6
1.14.13.39
1.14.14.47
Arginine
Arginine and proline metabolism
2.1.3.9
2.1.3.3
D-Arg & D-Orn metabolism
Citrate cycle
1.4.1.2
1.4.1.3
1.4.1.4
2.3.1.35
Ornithine
3.5.3.1
Urea-1-carboxylate
Urea
6.3.4.6
3.5.1.54
CO2
3.5.1.5
Excretion
2.6.1.1
2.6.1.2
2-Oxoglutarate
Glutamate
2.3.1.1
N-Acetyl-glutamate
2.7.2.8
N-Acetyl-glutamyl-P
1.2.1.38
N-Acetylglutamate semialdehyde
2.6.1.11
N-Acetyl-ornithine
3.5.1.14
3.5.1.16
ArgX
LysW-glutamate
ArgB
LysW-glutamyl-P
ArgC
LysW-L-glutamate semialdehyde
ArgD
LysW-ornithine
ArgE
00220 8/18/20
(c) Kanehisa Laboratories

eFigure 12

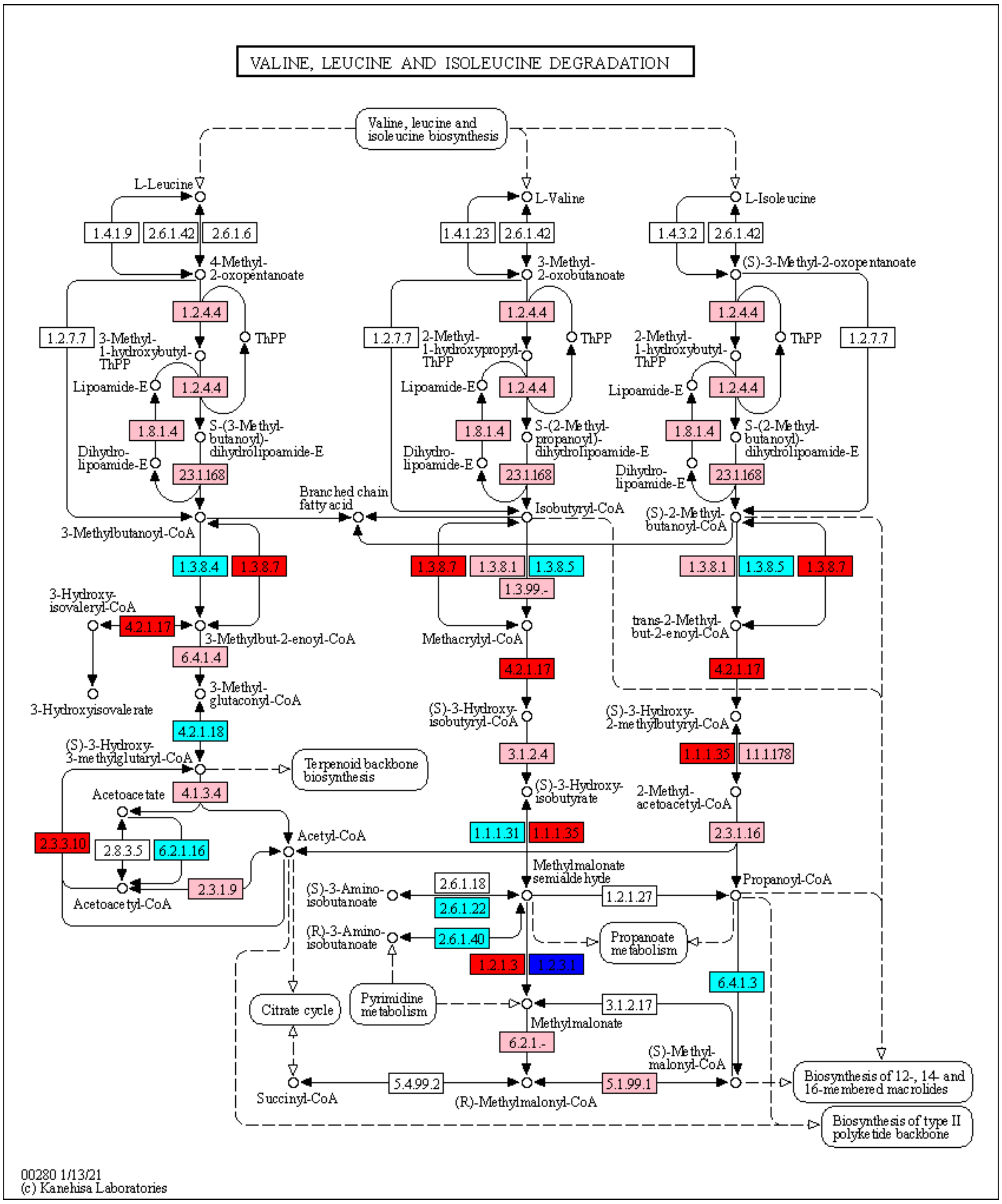
VALINE, LEUCINE AND ISOLEUCINE DEGRADATION
Valine, leucine and isoleucine biosynthesis
L-Leucine
L-Valine
L-Isoleucine
1.4.1.9
2.6.1.42
2.6.1.6
1.4.1.23
1.4.3.2
4-Methyl-2-oxopentanoate
3-Methyl-2-oxobutanoate
(S)-3-Methyl-2-oxopentanoate
1.2.4.4
ThPP
1.2.7.7
3-Methyl-1-hydroxybutyl-ThPP
2-Methyl-1-hydroxypropyl-ThPP
2-Methyl-1-hydroxybutyl-ThPP
Lipoamide-E
1.8.1.4
S-(3-Methylbutanoyl)-dihydrolipoamide-E
S-(2-Methylpropanoyl)-dihydrolipoamide-E
S-(2-Methylbutanoyl)-dihydrolipoamide-E
Dihydro-lipoamide-E
2.3.1.168
Branched chain fatty acid
3-Methylbutanoyl-CoA
Isobutyryl-CoA
(S)-2-Methylbutanoyl-CoA
1.3.8.4
1.3.8.7
1.3.8.1
1.3.8.5
1.3.99.-
3-Hydroxyisovaleryl-CoA
4.2.1.17
3-Methylbut-2-enoyl-CoA
Methacrylyl-CoA
trans-2-Methylbut-2-enoyl-CoA
6.4.1.4
3-Hydroxyisovalerate
3-Methylglutaconyl-CoA
4.2.1.18
(S)-3-Hydroxyisobutyryl-CoA
(S)-3-Hydroxy-2-methylbutyryl-CoA
(S)-3-Hydroxy-3-methylglutaryl-CoA
Terpenoid backbone biosynthesis
3.1.2.4
1.1.1.35
1.1.1.178
Acetoacetate
4.1.3.4
(S)-3-Hydroxyisobutyrate
2-Methylacetoacetyl-CoA
2.3.3.10
2.8.3.5
6.2.1.16
Acetyl-CoA
1.1.1.31
2.3.1.16
2.3.1.9
Acetoacetyl-CoA
Methylmalonate semialdehyde
Propanoyl-CoA
2.6.1.18
(S)-3-Amino-isobutanoate
2.6.1.22
1.2.1.27
(R)-3-Amino-isobutanoate
2.6.1.40
Propanoate metabolism
1.2.1.3
1.2.3.1
6.4.1.3
Citrate cycle
Pyrimidine metabolism
3.1.2.17
Methylmalonate
6.2.1.-
(S)-Methylmalonyl-CoA
Biosynthesis of 12-, 14- and 16-membered macrolides
Succinyl-CoA
5.4.99.2
(R)-Methylmalonyl-CoA
5.1.99.1
Biosynthesis of type II polyketide backbone
00280 1/13/21
(c) Kanehisa Laboratories

eFigure 13

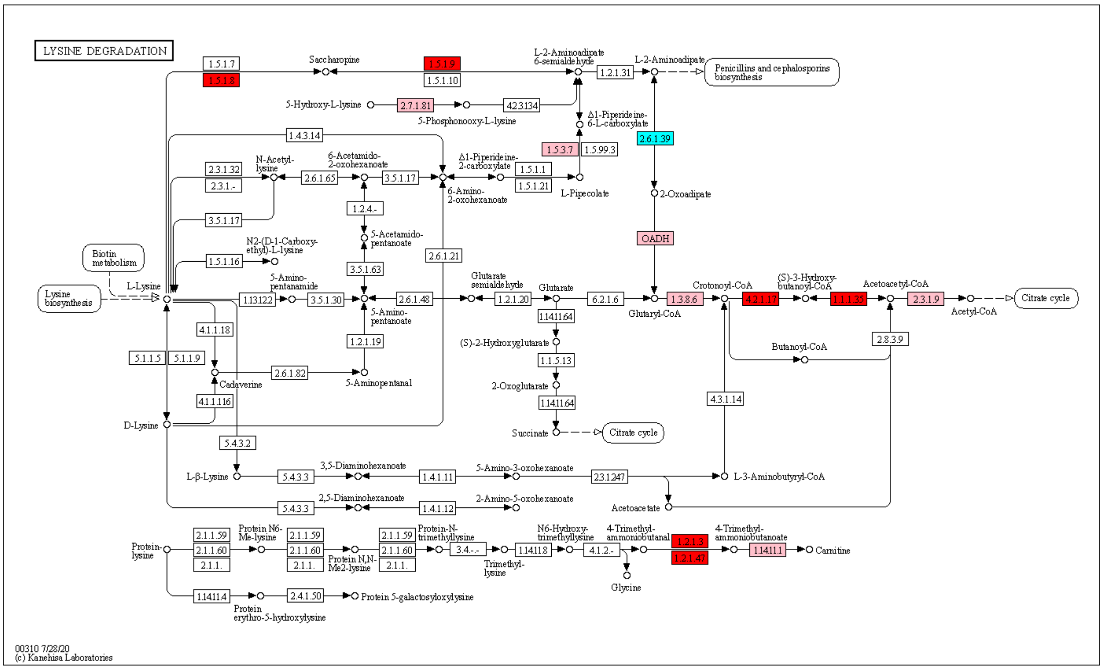
LYSINE DEGRADATION
1.5.1.7
1.5.1.8
Saccharopine
1.5.1.9
1.5.1.10
L-2-Aminoadipate 6-semialdehyde
1.2.1.31
L-2-Aminoadipate
Penicillins and cephalosporins biosynthesis
5-Hydroxy-L-lysine
2.7.1.81
5-Phosphonooxy-L-lysine
4.2.3.134
Δ1-Piperideine-6-L-carboxylate
1.5.3.7
1.5.99.3
2.6.1.39
1.4.3.14
2.3.1.32
2.3.1.-
N-Acetyl-lysine
2.6.1.65
6-Acetamido-2-oxohexanoate
3.5.1.17
Δ1-Piperideine-2-carboxylate
1.5.1.1
1.5.1.21
L-Pipecolate
6-Amino-2-oxohexanoate
2-Oxoadipate
3.5.1.17
1.2.4.-
5-Acetamido-pentanoate
N2-(D-1-Carboxy-ethyl)-L-lysine
1.5.1.16
Biotin metabolism
Lysine biosynthesis
L-Lysine
OADH
2.6.1.21
3.5.1.63
5-Amino-pentanamide
1.13.12.2
3.5.1.30
2.6.1.48
Glutarate semialdehyde
1.2.1.20
Glutarate
6.2.1.6
Glutaryl-CoA
1.3.8.6
Crotonoyl-CoA
4.2.1.17
(S)-3-Hydroxy-butanoyl-CoA
1.1.1.35
Acetoacetyl-CoA
2.3.1.9
Acetyl-CoA
Citrate cycle
5-Amino-pentanoate
1.14.11.64
4.1.1.18
1.2.1.19
(S)-2-Hydroxyglutarate
Butanoyl-CoA
2.8.3.9
5.1.1.5
5.1.1.9
2.6.1.82
Cadaverine
5-Aminopentanal
1.1.5.13
2-Oxoglutarate
4.1.1.116
1.14.11.64
4.3.1.14
D-Lysine
Succinate
Citrate cycle
5.4.3.2
L-β-Lysine
5.4.3.3
3,5-Diaminohexanoate
1.4.1.11
5-Amino-3-oxohexanoate
2.3.1.247
L-3-Aminobutyryl-CoA
5.4.3.3
2,5-Diaminohexanoate
1.4.1.12
2-Amino-5-oxohexanoate
Acetoacetate
Protein-lysine
2.1.1.59
2.1.1.60
2.1.1.
Protein N6-Me-lysine
2.1.1.59
2.1.1.60
2.1.1.
Protein N,N-Me2-lysine
2.1.1.59
2.1.1.60
2.1.1.
Protein-N-trimethyllysine
3.4.-.-
Trimethyl-lysine
1.14.11.8
N6-Hydroxy-trimethyllysine
4.1.2.-
Glycine
4-Trimethyl-ammoniobutanal
1.2.1.3
1.2.1.47
4-Trimethyl-ammoniobutanoate
1.14.11.1
Carnitine
1.14.11.4
Protein erythro-5-hydroxylysine
2.4.1.50
Protein 5-galactosyloxylysine
00310 7/28/20
(c) Kanehisa Laboratories

eFigure 14

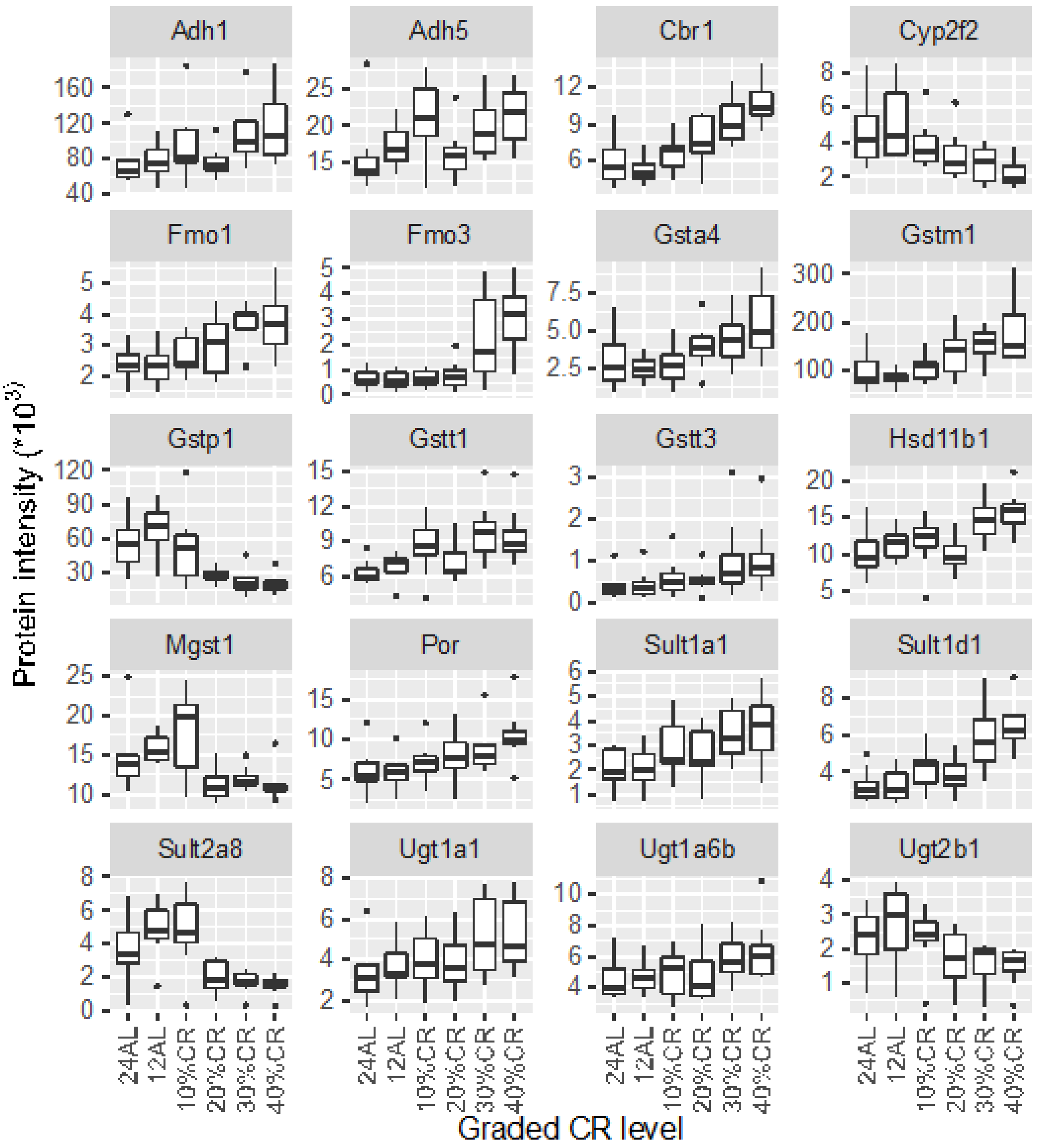
Adh1
Adh5
Cbr1
Cyp2f2
Fmo1
Fmo3
Gsta4
Gstm1
Gstp1
Gstt1
Gstt3
Hsd11b1
Mgst1
Por
Sult1a1
Sult1d1
Sult2a8
Ugt1a1
Ugt1a6b
Ugt2b1
Protein intensity (*10³)
24AL
12AL
10%CR
20%CR
30%CR
40%CR
Graded CR level

eFigure 15

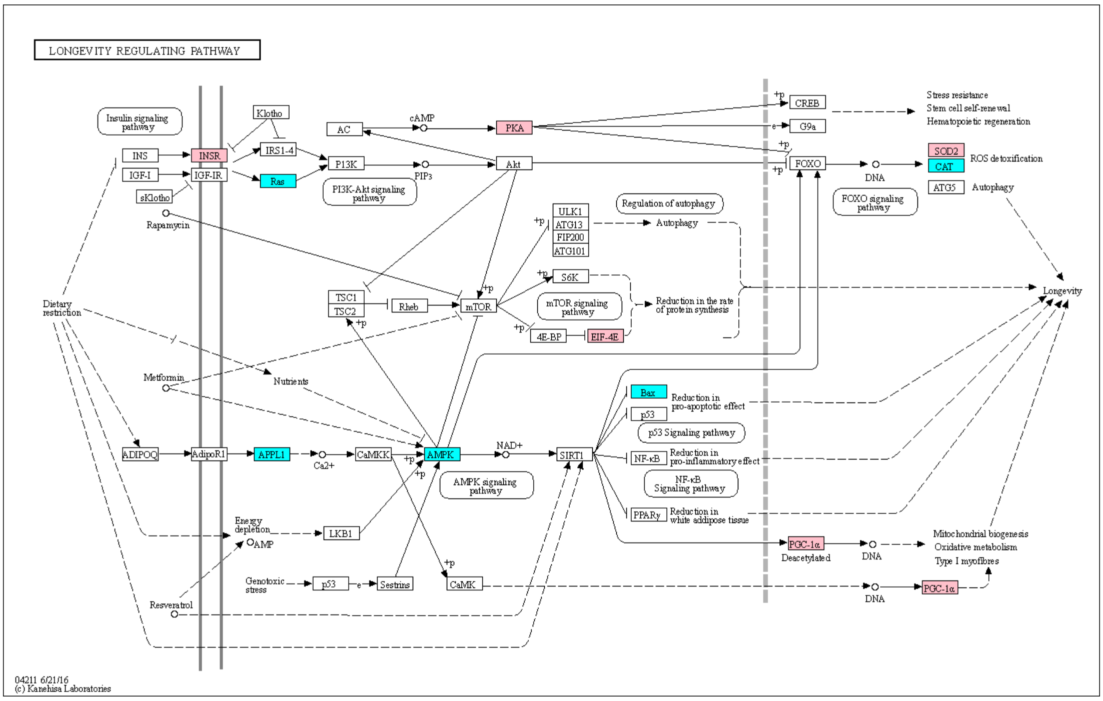
LONGEVITY REGULATING PATHWAY
Insulin signaling pathway
Klotho
INS
INSR
IGF-I
IGF-IR
sKlotho
IRS1-4
Ras
AC
cAMP
PKA
P13K
PIP3
Akt
PI3K-Akt signaling pathway
Rapamycin
TSC1
TSC2
Rheb
mTOR
ULK1
ATG13
FIP200
ATG101
Regulation of autophagy
Autophagy
S6K
mTOR signaling pathway
4E-BP
EIF-4E
Reduction in the rate of protein synthesis
CREB
G9a
FOXO
Stress resistance
Stem cell self-renewal
Hematopoietic regeneration
SOD2
CAT
ROS detoxification
ATG5
Autophagy
DNA
FOXO signaling pathway
Longevity
Dietary restriction
Metformin
Nutrients
ADIPOQ
AdipoR1
APPL1
Ca2+
CaMKK
AMPK
NAD+
SIRT1
AMPK signaling pathway
Bax
Reduction in pro-apoptotic effect
p53
p53 Signaling pathway
NF-κB
Reduction in pro-inflammatory effect
NF-κB Signaling pathway
PPARγ
Reduction in white addipose tissue
Energy depletion
AMP
LKB1
Genotoxic stress
p53
Sestrins
CaMK
Resveratrol
PGC-1α
Deacetylated
DNA
Mitochondrial biogenesis
Oxidative metabolism
Type I myofibres
DNA
PGC-1α
04211 6/21/16
(c) Kanehisa Laboratories

eFigure 16

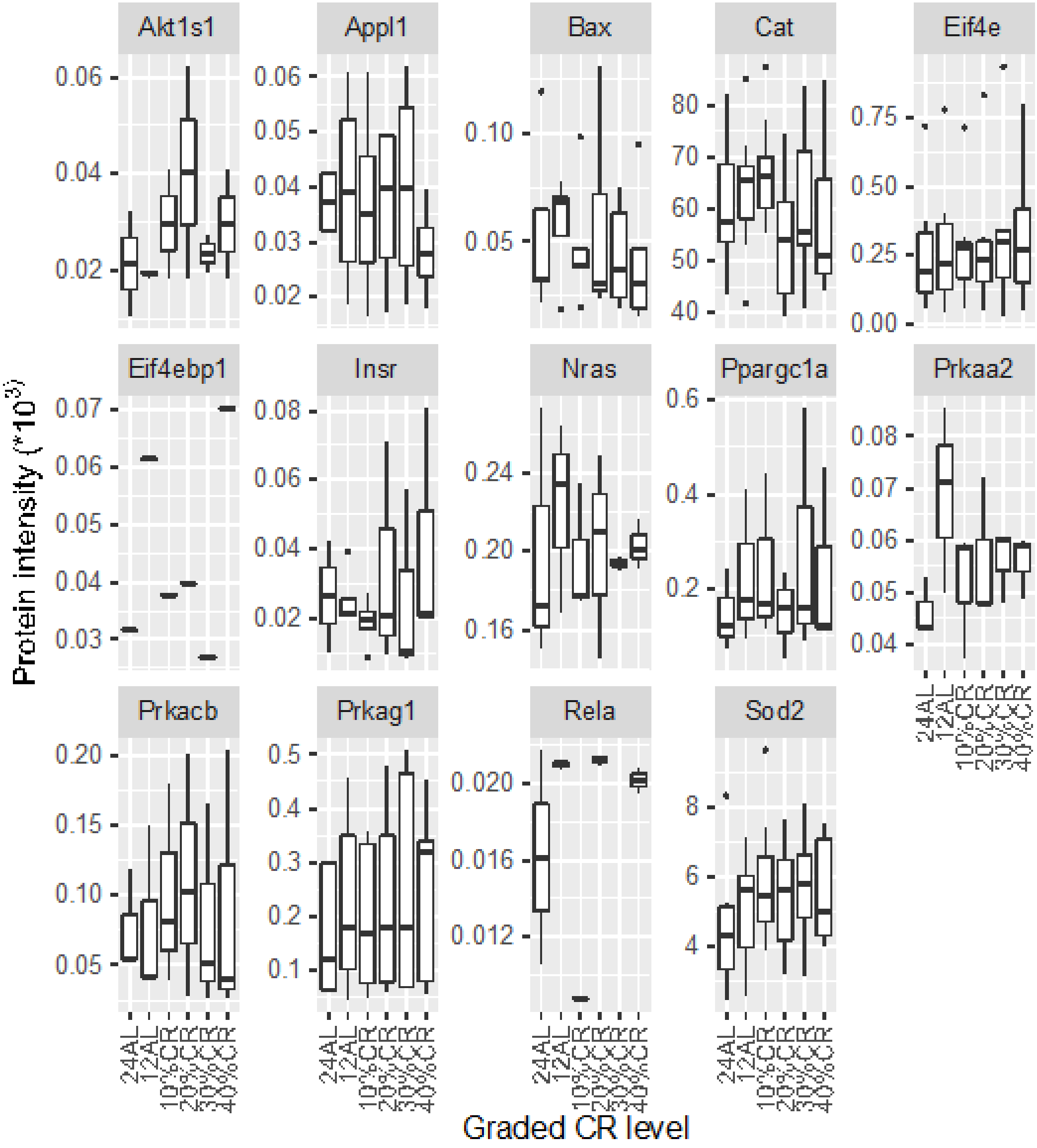

Akt1s1
Appl1
Bax
Cat
Eif4e
Eif4ebp1
Insr
Nras
Ppargc1a
Prkaa2
Prkacb
Prkag1
Rela
Sod2
Protein intensity (*10^3)
Graded CR level
24AL
12AL
10%CR
20%CR
30%CR
40%CR

eFigure 17

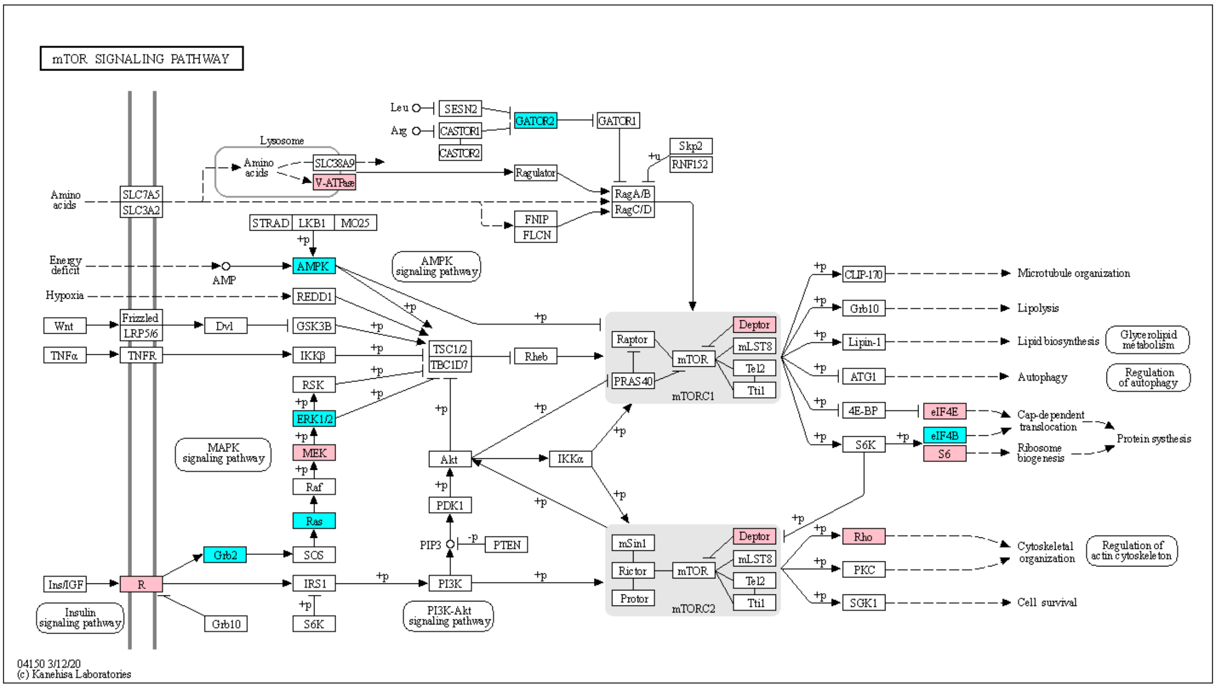

mTOR SIGNALING PATHWAY
Leu
Arg
SESN2
CASTOR1
CASTOR2
GATOR2
GATOR1
Skp2
RNF152
+u
Lysosome
Amino acids
SLC38A9
V-ATPase
Regulator
RagA/B
RagC/D
Amino acids
SLC7A5
SLC3A2
FNIP
FLCN
STRAD
LKB1
MO25
+p
Energy deficit
AMP
AMPK
AMPK signaling pathway
Hypoxia
REDD1
Wnt
Frizzled
LRP5/6
Dvl
GSK3B
TNFα
TNFR
IKKβ
RSK
ERK1/2
MEK
Raf
Ras
MAPK signaling pathway
TSC1/2
TBC1D7
Rheb
Raptor
mTOR
Deptor
mLST8
Tel2
Tti1
PRAS40
mTORC1
Akt
IKKα
PDK1
PIP3
-p
PTEN
PI3K
PI3K-Akt signaling pathway
Grb2
SOS
Ins/IGF
R
IRS1
S6K
Grb10
Insulin signaling pathway
mSin1
Rictor
Protor
mTORC2
CLIP-170
Microtubule organization
Grb10
Lipolysis
Lipin-1
Lipid biosynthesis
Glycerolipid metabolism
ATG1
Autophagy
Regulation of autophagy
4E-BP
eIF4E
Cap-dependent translocation
S6K
eIF4B
S6
Ribosome biogenesis
Protein systhesis
Rho
PKC
Cytoskeletal organization
Regulation of actin cytoskeleton
SGK1
Cell survival
04150 3/12/20
(c) Kanehisa Laboratories

eFigure 18

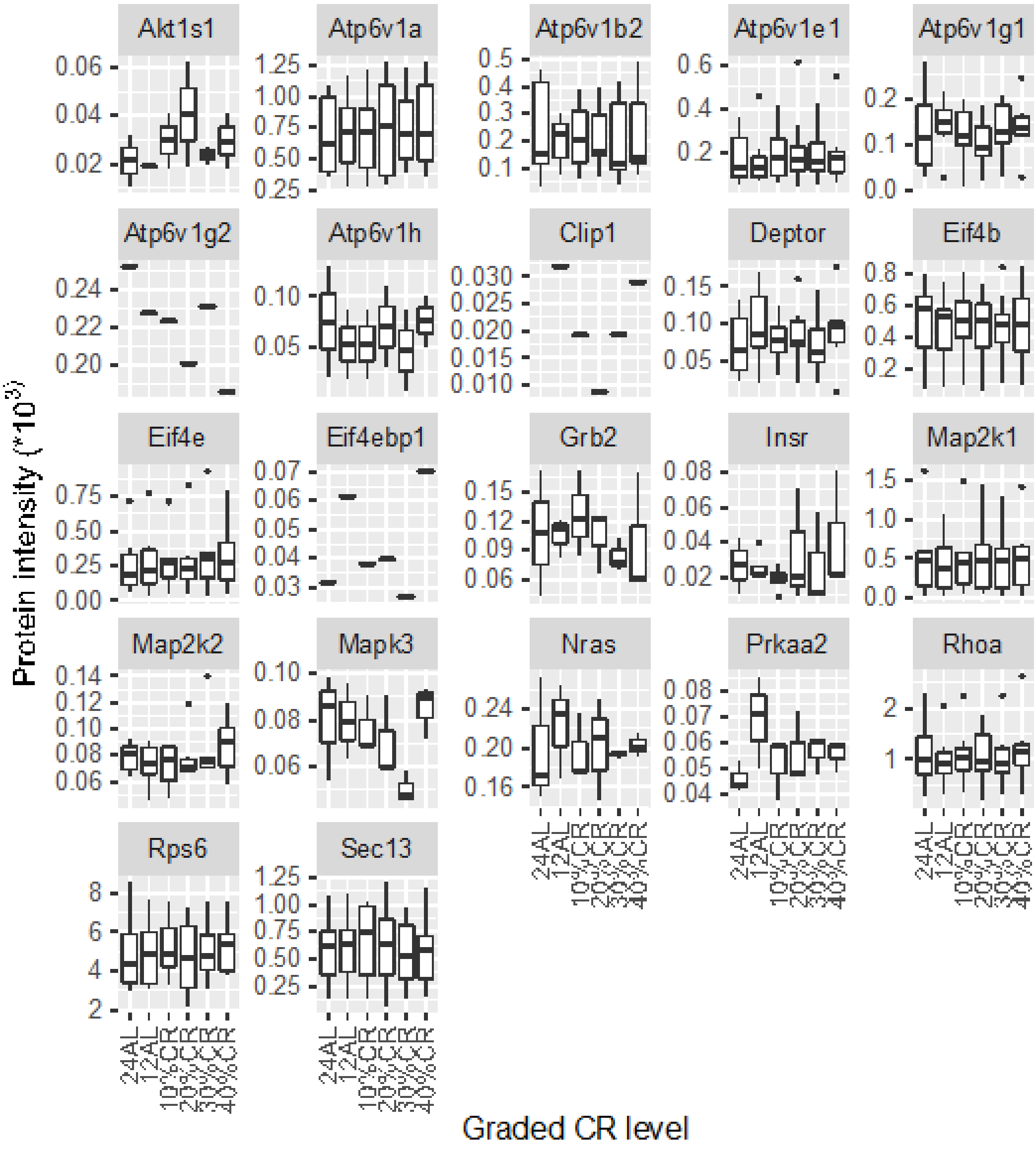
Akt1s1
Atp6v1a
Atp6v1b2
Atp6v1e1
Atp6v1g1
Atp6v1g2
Atp6v1h
Clip1
Deptor
Eif4b
Eif4e
Eif4ebp1
Grb2
Insr
Map2k1
Map2k2
Mapk3
Nras
Prkaa2
Rhoa
Rps6
Sec13
Protein intensity (*10^3)
24AL
12AL
10%CR
20%CR
30%CR
40%CR
Graded CR level

eFigure 19

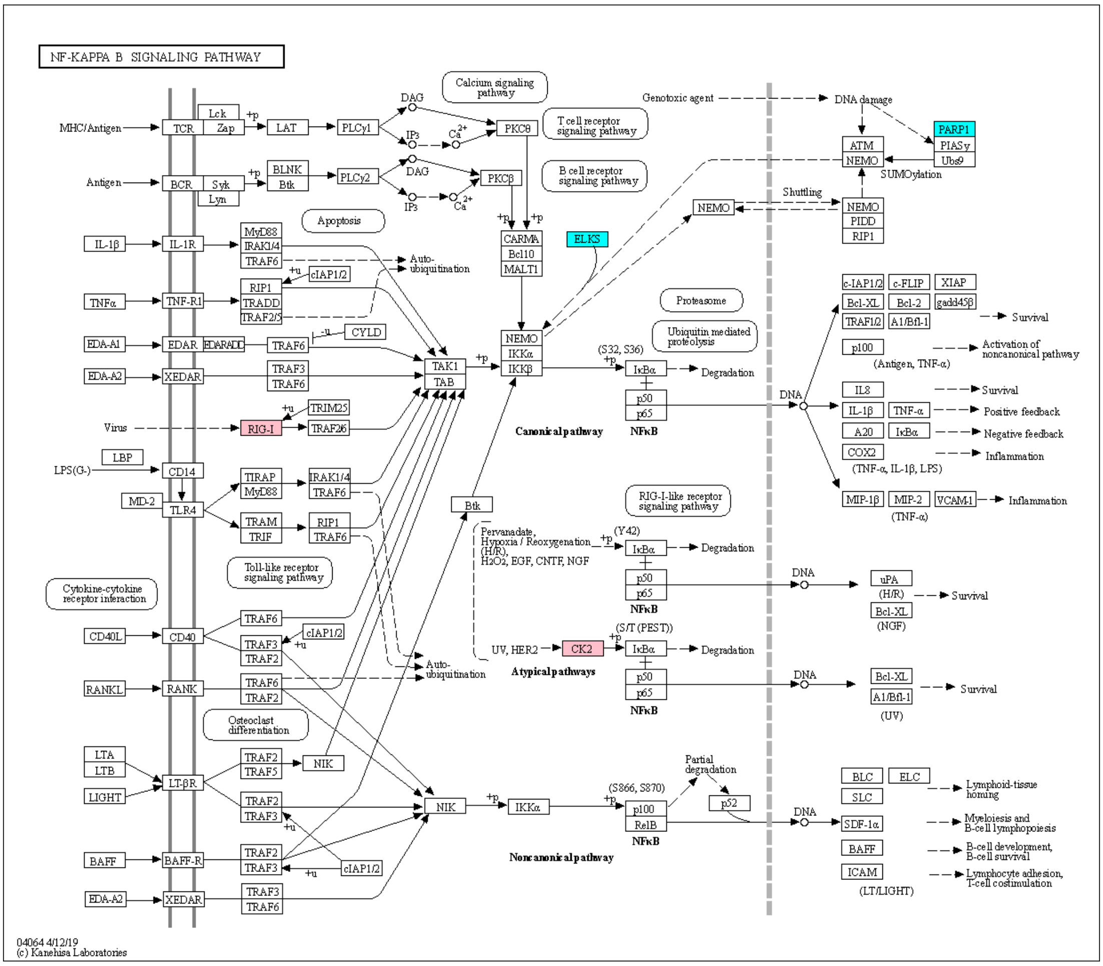
NF-KAPPA B SIGNALING PATHWAY
Calcium signaling pathway
T cell receptor signaling pathway
B cell receptor signaling pathway
Apoptosis
Proteasome
Ubiquitin mediated proteolysis
Toll-like receptor signaling pathway
Cytokine-cytokine receptor interaction
Osteoclast differentiation
RIG-I-like receptor signaling pathway
Canonical pathway
Atypical pathways
Noncanonical pathway
Genotoxic agent
DNA damage
PARP1
PIASy
Ubc9
ATM
NEMO
SUMOylation
Shuttling
PIDD
RIP1
ELKS
CK2
RIG-I
Survival
Activation of noncanonical pathway
Positive feedback
Negative feedback
Inflammation
Lymphoid-tissue homing
Myeloiesis and B-cell lymphopoiesis
B-cell development, B-cell survival
Lymphocyte adhesion, T-cell costimulation
Degradation
Auto-ubiquitination
Partial degradation
04064 4/12/19
(c) Kanehisa Laboratories

eFigure 20

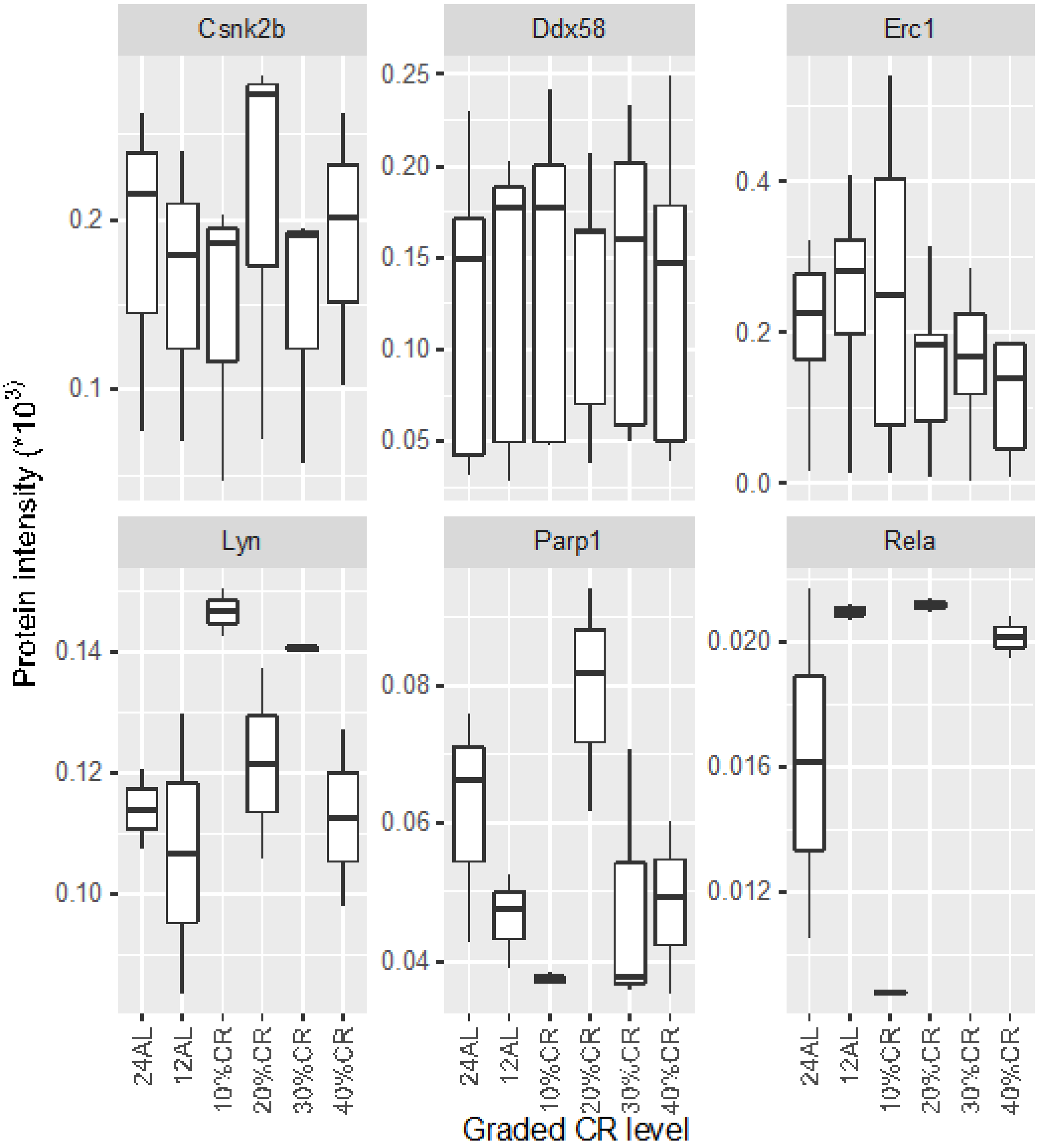

Csnk2b
Ddx58
Erc1
Lyn
Parp1
Rela
Protein intensity (*10^3)
Graded CR level
24AL
12AL
10%CR
20%CR
30%CR
40%CR
0.1
0.2
0.05
0.10
0.15
0.20
0.25
0.0
0.2
0.4
0.10
0.12
0.14
0.04
0.06
0.08
0.012
0.016
0.020

eFigure 21

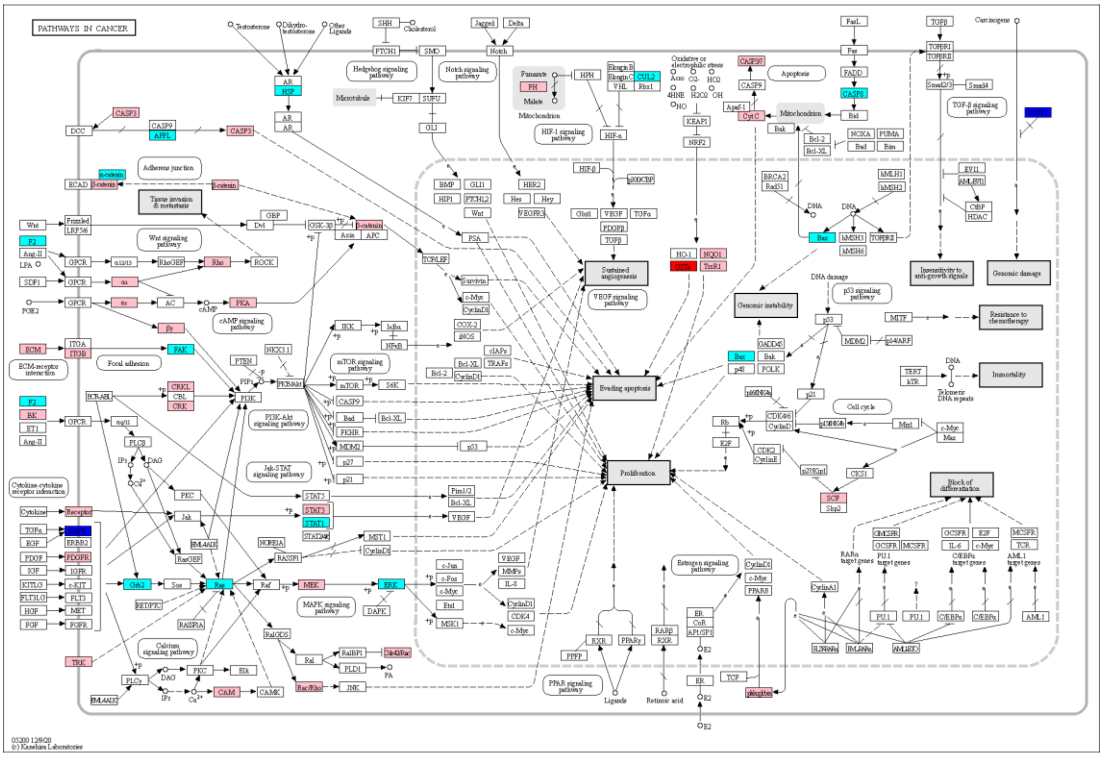

PATHWAYS IN CANCER
Hedgehog signaling pathway
Notch signaling pathway
Adherens junction
Tissue invasion & metastasis
Wnt signaling pathway
cAMP signaling pathway
Focal adhesion
ECM-receptor interaction
mTOR signaling pathway
PI3K-Akt signaling pathway
Jak-STAT signaling pathway
MAPK signaling pathway
Calcium signaling pathway
Cytokine-cytokine receptor interaction
HIF-1 signaling pathway
VEGF signaling pathway
Sustained angiogenesis
Evading apoptosis
Proliferation
Estrogen signaling pathway
PPAR signaling pathway
Genomic instability
p53 signaling pathway
Cell cycle
TGF-β signaling pathway
Insensitivity to anti-growth signals
Genomic damage
Resistance to chemotherapy
Immortality
Block of differentiation
Apoptosis
Mitochondrion
DNA damage
Carcinogens
05200 12/9/20
(c) Kanehisa Laboratories

eFigure 22

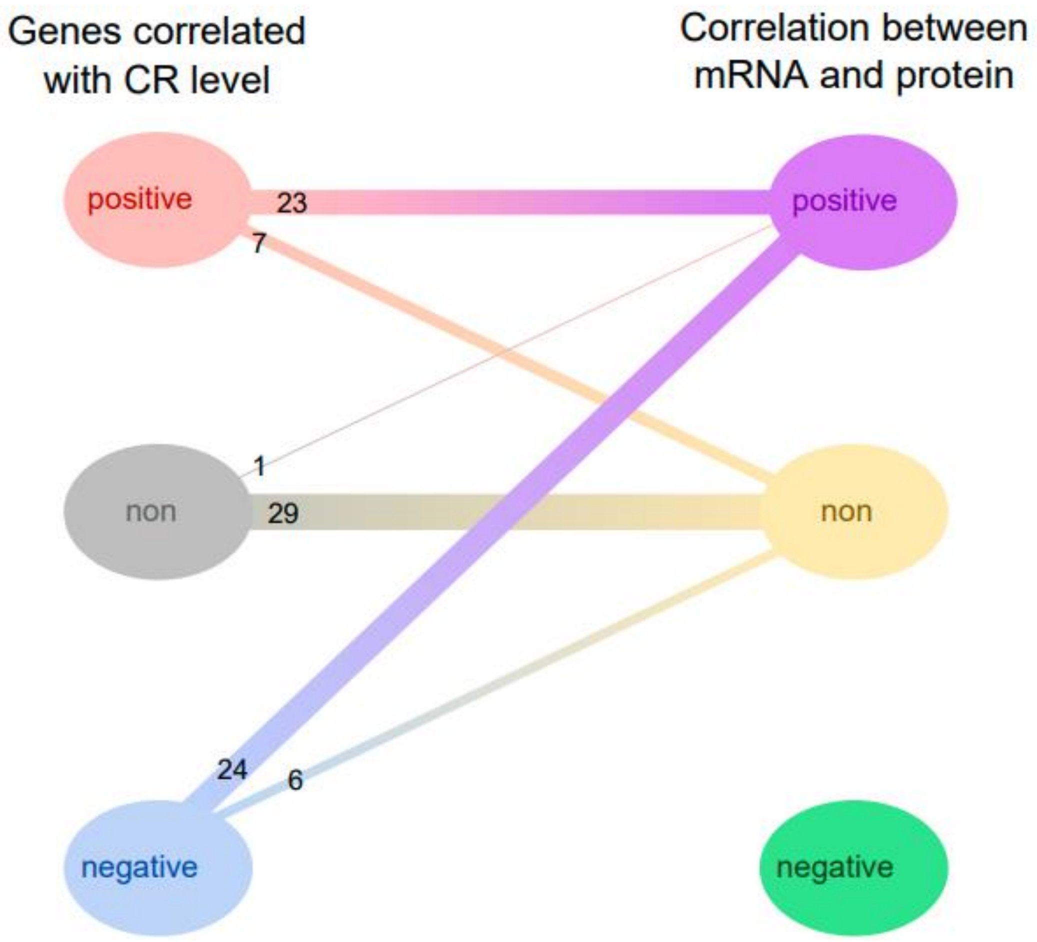

Genes correlated with CR level
Correlation between mRNA and protein
positive
23
7
positive
1
non
29
non
24
6
negative
negative

eFigure 23

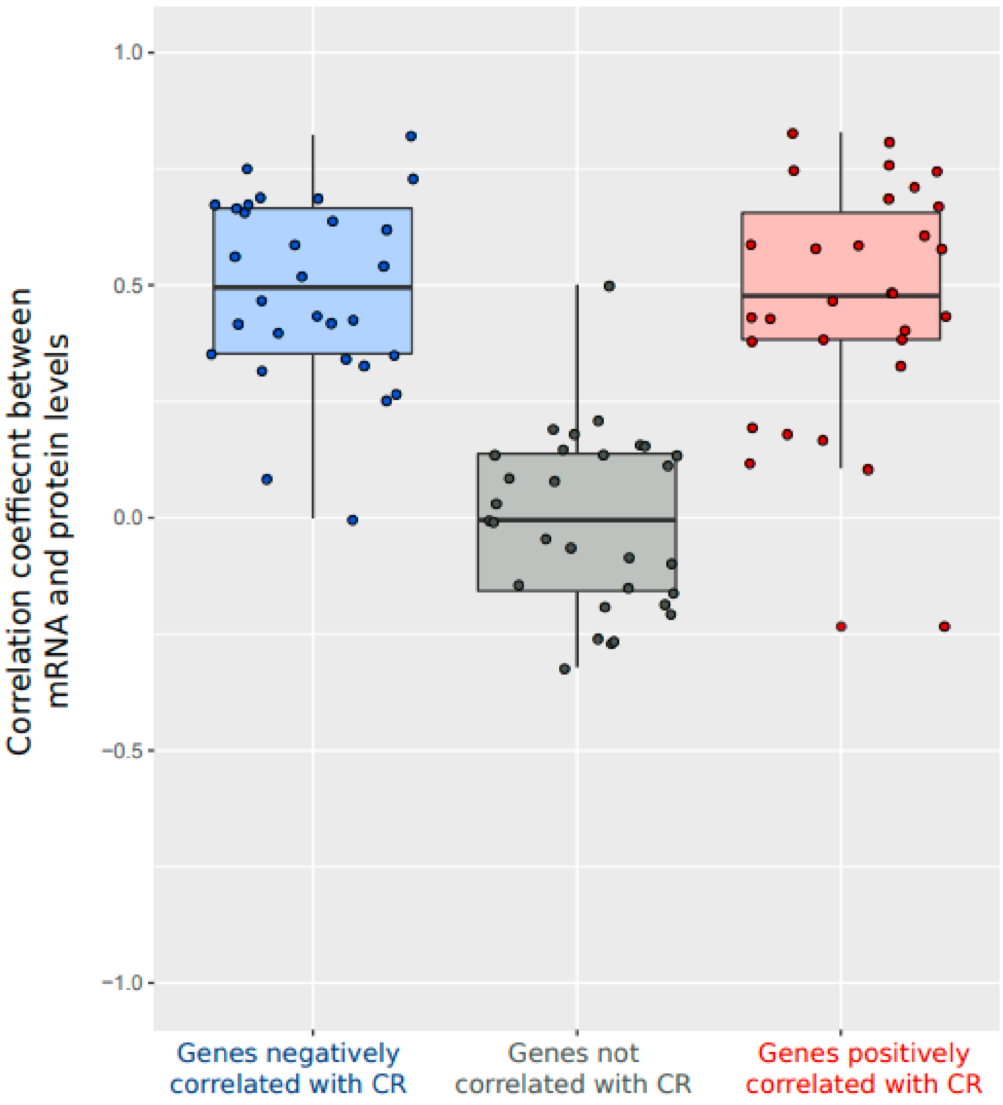

Correlation coeffiecnt between mRNA and protein levels
1.0
0.5
0.0
−0.5
−1.0
Genes negatively correlated with CR
Genes not correlated with CR
Genes positively correlated with CR
